# Clinical phenotypes of ANCA-associated vasculitis using ensemble clustering: prognostic and therapeutic insights from two Japanese cohorts

**DOI:** 10.1093/rheumatology/keag483

**Published:** 2026-09-17

**Authors:** Hironori Inoue, Takashi Kida, Kazuki Fujioka, Satoshi Omura, Takuya Yanagida, Yuki Shimada, Junya Kitai, Takahiro Seno, Masataka Kohno, Daiki Nakagomi, Yoshiyuki Abe, Makoto Wada, Naoho Takizawa, Atsushi Nomura, Yuji Kukida, Naoya Kondo, Hirosuke Takagi, Koji Endo, Shintaro Hirata, Naoto Azuma, Tohru Takeuchi, Shoichi Fukui, Kazuro Kamada, Ryo Yanai, Yusuke Matsuo, Yasuhiro Shimojima, Ryo Nishioka, Ryota Okazaki, Tomoaki Takata, Mayuko Moriyama, Ayuko Takatani, Yoshia Miyawaki, Tsuyoshi Shirai, Hiroaki Dobashi, Takafumi Ito, Isao Matsumoto, Toshihiko Takada, Toshiko Ito-Ihara, Nobuyuki Yajima, Takashi Kawaguchi, Hidekazu Ikeuchi, Izaya Nakaya, Kumiko Shimoyama, Koichi Amano, Tomomi Endo, Yusuke Ushio, Yoshinori Komagata, Taio Naniwa, Atsushi Kawakami, Tomoaki Higuchi, Kenji Nagasaka, Haruhito A Uchida, Naoto Tamura, Yutaka Kawahito, The J-CANVAS Study and JPVAS Cohort Study Collaborative Group Investigators, Masao Kikuchi, Hiroshi Akazawa, Tatsuya Atsumi, Tomonori Ishii, Tamihiro Kawakami, Hajime Kono, Yoshinori Matsumoto, Hiroaki Niiro, Natsuka Umezawa, Kotaro Matsumoto, Toshihiro Nanki, Ishizaki Jun, Kan Kishibe, Masashi Bando, Takao Fujii, Yasuhiro Maejima, Hajime Yoshifuji, Akinori Hara, Mamoru Ayusawa, Shuichi Ito, Mitsuyo Itabashi, Shinya Yamamoto, Masao Ogura, Toshiaki Usui, Kazunori Karasawa, Shoichiro Kanda, Takashi Kanda, Masanari Kodera, Miki Suzuki, Masaya Saito, Kiyoshi Sekiya, Naoko Nakano, Hiroko Nagafuchi, Hiroshi Nakajima, Motoshi Hattori, Kunihiro Ichinose, Masahiro Yamamura, Wako Yumura, Naoko Ishiguro, Maiko Tanaka, Shigeru Shibata, Ken-Ei Sada, Naotake Tsuboi, Naomi Iwata, Susumu Sakamoto, Masami Mizuno, Daisuke Kobayashi, Hiromichi Tamaki, Norihiro Kokudo, Aya Kawasaki, Eisuke Inoue, Fumihiko Matsuda, Yoshihisa Yamano, Shinji Kosugi, Motomu Hashimoto

**Affiliations:** Inflammation and Immunology, Graduate School of Medical Science, Kyoto Prefectural University of Medicine, Kyoto, Japan; Inflammation and Immunology, Graduate School of Medical Science, Kyoto Prefectural University of Medicine, Kyoto, Japan; Inflammation and Immunology, Graduate School of Medical Science, Kyoto Prefectural University of Medicine, Kyoto, Japan; Inflammation and Immunology, Graduate School of Medical Science, Kyoto Prefectural University of Medicine, Kyoto, Japan; Inflammation and Immunology, Graduate School of Medical Science, Kyoto Prefectural University of Medicine, Kyoto, Japan; Inflammation and Immunology, Graduate School of Medical Science, Kyoto Prefectural University of Medicine, Kyoto, Japan; Inflammation and Immunology, Graduate School of Medical Science, Kyoto Prefectural University of Medicine, Kyoto, Japan; Inflammation and Immunology, Graduate School of Medical Science, Kyoto Prefectural University of Medicine, Kyoto, Japan; Inflammation and Immunology, Graduate School of Medical Science, Kyoto Prefectural University of Medicine, Kyoto, Japan; Department of Rheumatology, University of Yamanashi Hospital, Chuo, Japan; Department of Internal Medicine and Rheumatology, Juntendo University, Tokyo, Japan; Center for Rheumatic Disease, Japanese Red Cross Society Kyoto Daiichi Hospital, Kyoto, Japan; Department of Rheumatology, Chubu Rosai Hospital, Nagoya, Japan; Immuno-Rheumatology Center, St. Luke’s International Hospital, Tokyo, Japan; Department of Rheumatology, Japanese Red Cross Society Kyoto Daini Hospital, Kyoto, Japan; Department of Nephrology, Kyoto Katsura Hospital, Kyoto, Japan; Department of Hematology and Rheumatology, Kagoshima University Hospital, Kagoshima, Japan; Department of General Internal Medicine, Tottori Red Cross Hospital, Tottori, Japan; Department of Clinical Immunology and Rheumatology, Hiroshima University Hospital, Hiroshima, Japan; Department of Diabetes, Endocrinology and Clinical Immunology, Hyogo Medical University School of Medicine, Nishinomiya, Japan; Department of Internal Medicine (IV), Osaka Medical and Pharmaceutical University, Takatsuki, Japan; Department of Immunology and Rheumatology, Division of Advanced Preventive Medical Sciences, Nagasaki University Graduate School of Biomedical Sciences, Nagasaki, Japan; Department of Rheumatology, Endocrinology and Nephrology, Faculty of Medicine and Graduate School of Medicine, Hokkaido University, Sapporo, Japan; Division of Rheumatology, Department of Medicine, Showa Medical University School of Medicine, Tokyo, Japan; Department of Rheumatology, Tokyo Kyosai Hospital, Tokyo, Japan; Department of Medicine (Neurology and Rheumatology), Shinshu University School of Medicine, Matsumoto, Japan; Department of Rheumatology, Fukushima Medical University School of Medicine, Fukushima, Japan; Department of Nephrology and Rheumatology, Graduate School of Medical Science, Kanazawa University, Kanazawa, Japan; Division of Respiratory Medicine and Rheumatology, Department of Multidisciplinary Internal Medicine, Faculty of Medicine, Tottori University, Yonago, Japan; Division of Gastroenterology and Nephrology, Faculty of Medicine, Tottori University, Yonago, Japan; Department of Rheumatology, Shimane University Faculty of Medicine, Izumo, Japan; Rheumatic Disease Center, Sasebo Chuo Hospital, Sasebo, Japan; Department of Public Health, Nagasaki University Graduate School of Biomedical Sciences, Nagasaki, Japan; Department of Nephrology, Rheumatology, Endocrinology and Metabolism, Okayama University Graduate School of Medicine, Dentistry and Pharmaceutical Sciences, Okayama, Japan; Department of Rheumatology, Tohoku University Hospital, Sendai, Japan; Division of Hematology, Rheumatology and Respiratory Medicine, Department of Internal Medicine, Faculty of Medicine, Kagawa University, Miki, Japan; Division of Nephrology, Department of Internal Medicine, Teikyo University Chiba Medical Center, Ichihara, Japan; Department of Rheumatology, Institute of Medicine, University of Tsukuba, Tsukuba, Japan; Department of General Medicine, Shirakawa Satellite for Teaching And Research (STAR) Fukushima Medical University, Shirakawa, Japan; The Clinical and Translational Research Center, University Hospital, Kyoto Prefectural University of Medicine, Kyoto, Japan; Division of Rheumatology, Department of Medicine, Showa Medical University School of Medicine, Tokyo, Japan; Department of Clinical Assessment, Tokyo University of Pharmacy and Life Sciences, Hachioji, Japan; Department of Nephrology and Rheumatology, Gunma University Graduate School of Medicine, Maebashi, Japan; Department of Nephrology and Rheumatology, Iwate Prefectural Central Hospital, Morioka, Japan; Division of Rheumatology, Internal Medicine III, Hamamatsu University School of Medicine, Hamamatsu, Japan; Department of Rheumatology and Clinical Immunology, Saitama Medical Centre, Saitama Medical University, Kawagoe, Japan; Department of Nephrology and Dialysis, Medical Research Institute Kitano Hospital, PIIF Tazuke-Kofukai, Osaka, Japan; Division of Hematology, Rheumatology and Respiratory Medicine, Department of Internal Medicine, Faculty of Medicine, Kagawa University, Miki, Japan; Department of Nephrology and Rheumatology, Kyorin University School of Medicine, Mitaka, Japan; Department of Respiratory Medicine, Allergy and Clinical Immunology, Nagoya City University Graduate School of Medical Sciences, Nagoya, Japan; DEJIMA Infectious Disease Research Alliance (DIDA), Nagasaki University, Nagasaki, Japan; Clinical Research Center Deputy Director, Nagasaki University Hospital, Nagasaki, Japan; Faculty of Biomedical Sciences, Nagasaki University, Nagasaki, Japan; Division of Rheumatology, Department of Internal Medicine, Tokyo Women’s Medical University School of Medicine, Tokyo, Japan; Department of Rheumatology, Ome Medical Center, Ome, Japan; Department of Rheumatology, Institute of Science Tokyo, Tokyo, Japan; Division of Kidney, Diabetes and Endocrine Diseases, Okayama University Medical Development Field, Okayama, Japan; Department of Internal Medicine and Rheumatology, Juntendo University, Tokyo, Japan; Inflammation and Immunology, Graduate School of Medical Science, Kyoto Prefectural University of Medicine, Kyoto, Japan

**Keywords:** ANCA-associated vasculitis, granulomatosis with polyangiitis, microscopic polyangiitis, cluster, mortality, J-CANVAS, JPVAS

## Abstract

**Objectives:**

Clinical heterogeneity in ANCA-associated vasculitis (AAV) is not fully captured by conventional disease subtypes or ANCA serotypes. We aimed to identify data-driven, clinically meaningful disease phenotypes based on patterns of organ involvement in microscopic polyangiitis (MPA) and granulomatosis with polyangiitis (GPA), and to evaluate their prognostic and therapeutic implications.

**Methods:**

We conducted a multicentre retrospective cohort study using two nationwide Japanese registries (J-CANVAS as derivation; JPVAS as validation). Ensemble clustering was applied to high-dimensional organ involvement data derived from the Birmingham Vasculitis Activity Score and additional clinically relevant manifestations. The resulting clusters were replicated using classification and regression tree (CART) across the derivation and validation cohorts, and associations between cluster memberships and clinical outcomes (overall survival and relapse), as well as response to induction therapy, were evaluated.

**Results:**

Among 726 patients with newly diagnosed MPA or GPA, ensemble clustering identified four reproducible clinical phenotypes: (a) renal-dominant without interstitial lung disease (ILD); (b) renal-dominant with ILD; (c) systemic multi-organ and (d) ear, nose and throat (ENT)-dominant. CART reproduced these clusters with good concordance. Overall survival and relapse incidence differed across clusters. The ENT-dominant cluster demonstrated favourable survival but a higher relapse risk and showed a trend towards improved relapse-free survival with rituximab compared with CYC, even among MPO-ANCA-positive patients.

**Conclusion:**

Organ involvement-based ensemble clustering identifies clinically meaningful and reproducible AAV phenotypes with distinct prognostic and therapeutic profiles. Integrating phenotypic patterns with ANCA serotype may enhance risk stratification and support more individualized management strategies in AAV.

## Graphical Abstract

**Graphical Abstract:**
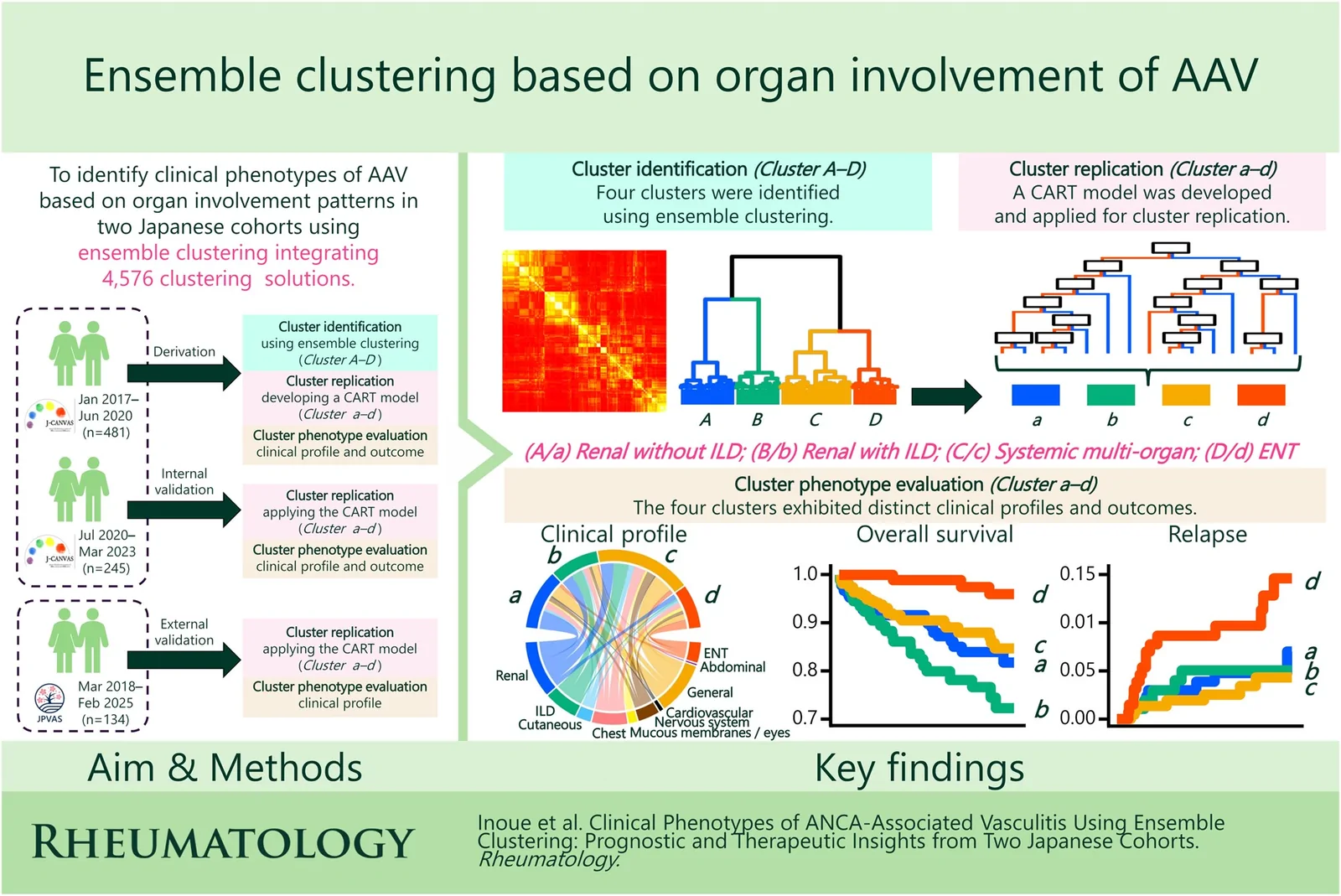

*Rheumatology* key messages

- Four distinct clinical phenotypes based on patterns of organ involvement were identified in patients with AAV.
- These phenotypes were associated with differences in overall survival and relapse risk.
- Organ involvement-based phenotyping captures clinical heterogeneity in addition to conventional serologic classifications.

## Introduction

ANCA-associated vasculitis (AAV) is a systemic necrotizing vasculitis affecting small- to medium-sized vessels and is characterized by ANCA positivity [1]. The 2012 Chapel Hill Consensus Conference (CHCC) defines AAV as comprising microscopic polyangiitis (MPA), granulomatosis with polyangiitis (GPA) and eosinophilic GPA [2]. The 2022 American College of Rheumatology/European Alliance of Associations for Rheumatology (ACR/EULAR) classification criteria have refined these entities by integrating clinical manifestations with ANCA serotypes [3–5]. Nevertheless, substantial clinical heterogeneity remains within and across conventional disease subtypes.

ANCA serotypes, MPO-ANCA and PR3-ANCA, are associated with genetic background, prognosis and treatment response [6, 7], but do not fully explain the diversity of clinical manifestations and outcomes. In clinical practice, the type and extent of organ involvement form the basis of disease activity and severity assessments such as the Birmingham Vasculitis Activity Score (BVAS) and the Five-Factor Score (FFS) [8, 9]. Therapeutic decision-making is also driven by patterns of organ involvement [1, 10].

Several clustering studies have explored clinical phenotypes in AAV; however, analyses based on less granular data may not adequately capture organ involvement patterns. A comprehensive, organ-based approach may identify clinically meaningful subtypes beyond traditional classifications. Moreover, an approach that defines clinical phenotypes based on organ involvement patterns is applicable to ANCA-negative or serologically unclassifiable cases, facilitating integrated stratification based on both ANCA serotypes and clinical features and providing a more comprehensive representation of disease heterogeneity.

To address this, we applied ensemble clustering to high-dimensional organ involvement data derived from BVAS [8], supplemented with additional manifestations. Ensemble clustering enhances robustness and reproducibility by aggregating results across multiple clustering algorithms and dimensional-reduction techniques, and is particularly well suited for detecting latent structures in high-dimensional data [11]. Accordingly, we aimed to identify reproducible clinical phenotypes based on organ involvement patterns and to evaluate their prognostic and therapeutic implications.

## Methods

### Study design and cohorts

**Figure 1:**
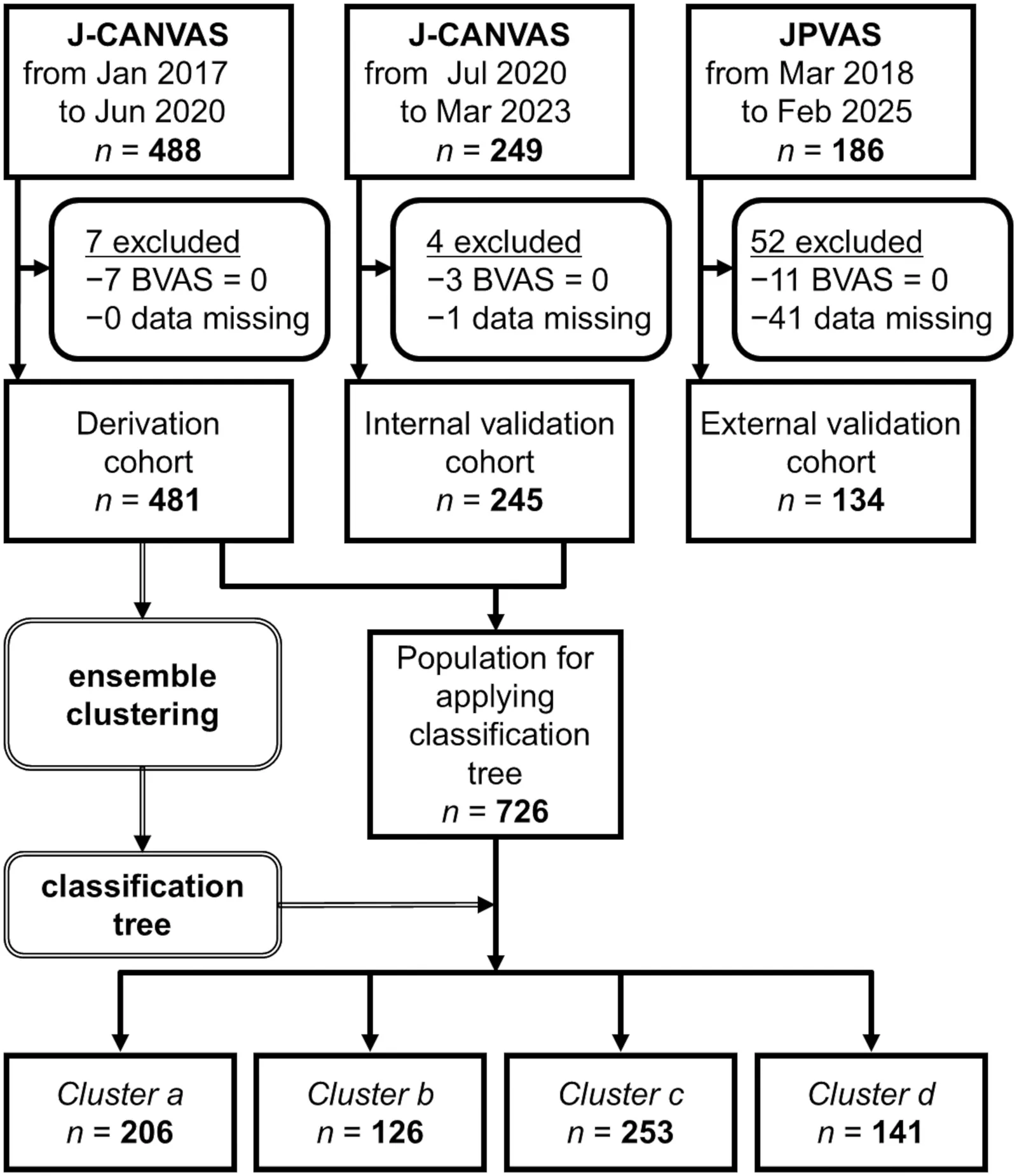
Flow diagram of patients in the study. Ensemble clustering was applied to the derivation cohort. A CART model was validated in both the internal and external cohorts. Clinical outcomes of cluster membership were assessed in the derivation and internal validation cohorts. BVAS: Birmingham vasculitis activity score (v.3); CART: classification and regression tree; J-CANVAS: Japan Collaborative Registry of ANCA-Associated Vasculitis; JPVAS: Japan Research Committee of the Ministry of Health, Labour, and Welfare for Intractable Vasculitis

We conducted a multicentre, registry-based retrospective observational study to identify and validate baseline organ involvement-based clinical phenotypes of AAV (Fig. 1). Data were obtained from two nationwide Japanese registries: the Japan Collaborative Registry of ANCA-Associated Vasculitis (J-CANVAS) study and the Japan Research Committee of the Ministry of Health, Labour, and Welfare (MHLW) for Intractable Vasculitis (JPVAS) prospective cohort study. Diagnoses and classifications in J-CANVAS were established according to the 2012 CHCC definitions and the European Medicines Agency algorithm [2, 12], whereas those in JPVAS were based on MHLW criteria [13] integrating clinical features, ANCA testing and imaging or histopathologic findings.

The derivation cohort comprised patients from J-CANVAS newly diagnosed with MPA or GPA between January 2017 and June 2020. The internal validation cohort included patients from J-CANVAS newly diagnosed between July 2020 and March 2023, and the external validation cohort included patients from JPVAS newly diagnosed between March 2018 and February 2025. Patients with a BVAS score of 0 at diagnosis or missing serum creatinine data were excluded.

Ensemble clustering was applied to the derivation cohort to detect organ involvement-based phenotypic clusters. A classification and regression tree (CART) model was trained to replicate the ensemble-derived clusters and subsequently validated in both the internal and external validation cohorts to evaluate reproducibility. In addition, associations between cluster membership and clinical outcomes were assessed in the derivation and internal validation cohorts.

### Data collection

Both registries collected standardized clinical and laboratory data. In J-CANVAS, data were extracted from medical records and entered into an electronic data capture system (Viedoc, PCG Solutions, Uppsala, Sweden). In JPVAS, data were extracted from medical records and standardized investigation forms for MHLW-designated intractable diseases, then entered into the electronic data capture system of the Rare Disease Data Registry of Japan. Baseline variables included: (1) patient and disease characteristics (age, sex, smoking status, diagnosis date and disease subtype); (2) organ involvement data derived from BVAS version 3 items (general; cutaneous; mucous membrane and eyes; ear, nose and throat [ENT]; chest; cardiovascular; abdominal; renal and nervous system) and additional clinically relevant manifestations informed by the 2022 ACR/EULAR classification criteria [3–5] (interstitial lung diseases (ILD) [confirmed by imaging], hypertrophic pachymeningitis, mastoiditis, nasal chondritis and auricular chondritis); (3) serologic and laboratory data (MPO-ANCA and PR3-ANCA status, and serum creatinine); (4) comorbidities (hypertension, diabetes, chronic kidney disease (CKD) [defined as a persistent estimated glomerular filtration rate (eGFR) < 60 prior to the diagnosis of AAV] [14], lung disease, cardiac disease, cerebrovascular disease and malignancy) and (5) induction therapy (use of intravenous methylprednisolone [IVMP], rituximab [RTX], intravenous CYC or oral CYC).

Clinical outcomes included overall survival and relapse. Relapse was defined as a severe, organ-threatening or life-threatening increase in disease activity [15, 16].

### Cluster identification via ensemble clustering

To derive stable and reproducible clinical phenotypes, we applied an ensemble clustering framework integrating multiple variable representations, distance metrics and clustering algorithms, following the approach of Ronan *et al.* [11].

### Variable preprocessing

Sixty-one candidate variables were extracted from BVAS (56 items) and additional manifestations (ILD, hypertrophic pachymeningitis, mastoiditis, nasal chondritis, auricular chondritis). ‘Respiratory failure’ was removed because it reflected end-stage compromise rather than specific organ pathology. Closely related items were merged, and five variable sets with increasing levels of dimensional reduction were constructed (Supplementary Table S1):

- Variable set 1: 53 binary variables created by merging nearly identical clinical manifestations.
- Variable set 2: 32 binary variables created by further grouping clinically similar manifestations.
- Variable set 3: 13 binary variables, including the nine BVAS organ systems.
- Variable sets 4 and 5: principal component analyses of variable set 1, retaining the top 22 and 10 continuous components, respectively, which explained >90% and ∼65% of total variance.

### Clustering procedures

For binary variable sets, simple matching coefficient, Jaccard and Pearson’s *φ* distances were used; for continuous datasets, Euclidean, maximum, Manhattan, Canberra and correlation distances were applied. Each distance matrix was subjected to five hierarchical agglomerative methods (Ward’s, complete-linkage, single-linkage, group-average and centroid). Invalid combinations were excluded, yielding 72 valid dendrograms. Each dendrogram was partitioned into 3–10 clusters to generate 576 co-occurrence matrices, which were averaged to form the hierarchical ensemble matrix.

In parallel, *k*-means clustering (100 replicates per dataset, clusters = 3–10) produced 4000 additional co-occurrence matrices; their mean yielded the *k*-means ensemble matrix. The hierarchical and *k*-means ensemble matrices were then integrated to create the final co-occurrence matrix for subsequent cluster extraction.

### Determination of optimal cluster number

Cluster stability and validity were evaluated using the NbClust package [17]. Of the 30 indices provided, 20 were interpretable, and most of them supported a four-cluster solution. Visual inspection of the dendrogram and co-occurrence heatmap confirmed four distinct clusters (Clusters A–D).

### Cluster visualization and variable importance

To facilitate clinical interpretation of the derived clusters, relationships between each cluster and patterns of organ involvement were visualized using chord diagrams. A multiclass eXtreme Gradient Boosting (XGBoost) model was then trained using variable set 1 (53 items) as predictors and the ensemble-derived cluster assignments as labels. Feature importance was quantified using SHapley Additive exPlanations (SHAP) values, and beeswarm plots were used to identify variables defining each cluster. The top 20 variables with non-zero SHAP values were selected for the subsequent CART analysis.

### Cluster replication and validation

To enable cluster replication in independent populations, a CART model was constructed using the 20 variables identified above. The model employed Gini impurity as the splitting criterion. Two pruning strategies were independently performed and compared: (1) cost-complexity optimization and (2) minimization of test-set misclassification. Concordance between ensemble-derived and CART-derived cluster assignments was quantified using the adjusted Rand index (ARI) [18]. The finalized CART model was applied to the derivation cohort as well as the internal and external validation cohorts to assign patients to Clusters a–d, corresponding to ensemble-derived Clusters A–D.

### Clinical evaluation of cluster validity

To assess the clinical relevance of clustering, clinical outcomes (overall survival and relapse) were compared across CART-derived clusters. Subgroup analyses stratified by ANCA serotypes and disease subtypes, as well as evaluations of adjusted survival curves and adjusted cumulative incidence of relapse, were also conducted. Furthermore, within each CART-derived cluster, treatment responses were evaluated by comparing relapse-free survival between induction regimens (RTX, CYC or others) initiated within 2 weeks of diagnosis.

### Statistical analysis

Continuous variables were summarized as median (interquartile range [IQR]), and categorical variables as *n* (%). Overall survival and relapse-free survival were compared using the log-rank test, while the cumulative incidence of relapse was evaluated using the Gray test with death treated as a competing risk. Adjusted survival curves based on the corrected group prognosis method [19] were generated using Cox proportional hazards models that included the following covariates: age, sex, smoking status, disease subtype, ANCA serotype, baseline eGFR <30 mL/min/1.73 m^2^ and comorbidities such as hypertension, diabetes, CKD, lung disease, cardiac disease, cerebrovascular disease and malignancy. Using the same set of covariates, direct adjusted cumulative incidence curves for relapse were then estimated using Fine-Gray proportional hazards models [20]. All analyses were conducted using R software v4.3.1 (R Foundation for Statistical Computing, Vienna, Austria).

### Patient and public involvement statement

Patients and/or the public were not involved in the design, conduct, reporting or dissemination plans of this research.

### Ethics

This study complied with the principles of the Declaration of Helsinki. Because of the use of only anonymized data, written informed consent was not obtained from each patient. We provided an opportunity for patients to opt out of the study. The ethics committee of Kyoto Prefectural University of Medicine approved the protocol (Approval No. ERB-C-3579).

## Results

### Derivation, internal validation and external validation cohorts

In J-CANVAS, 737 patients were newly diagnosed with MPA or GPA between January 2017 and March 2023. After excluding 11 patients, 726 patients (481 in the derivation cohort and 245 in the internal validation cohort) were included in this analysis (Fig. 1). Clinical profiles are summarized in Supplementary Table S2.

In JPVAS, 186 patients were newly diagnosed with MPA or GPA between March 2018 and February 2025. After excluding 52 patients, 134 patients were assigned to the external validation cohort (Fig. 1). Clinical profiles are summarized in Supplementary Table S3.

### Ensemble clustering and cluster interpretation

**Figure 2:**
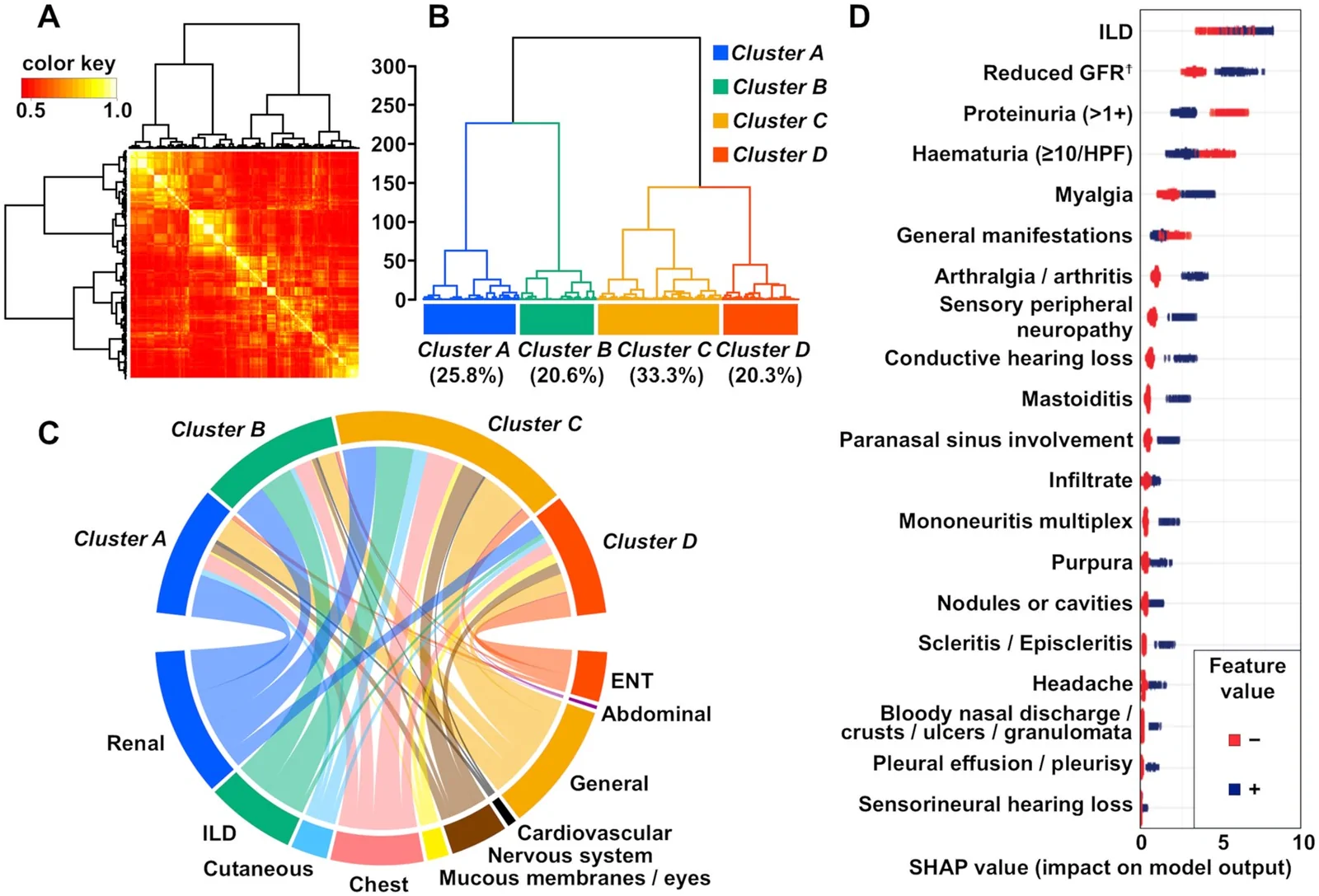
Ensemble clustering on organ involvement of patients with MPA or GPA. (**A**) Co-occurrence matrix heatmap demonstrating clustering of organ involvement. (**B**) Dendrogram generated from the heatmap. Four clusters were identified (Clusters A–D). (**C**) Chord diagram illustrating the associations of the clusters with BVAS organ systems and ILD. Band widths represent the cumulative number of organ involvements for each patient. (**D**) The top 20 variables contributing to the XGBoost model, which was developed to perform multiclass classification. ☨defined as serum creatinine ≥125 μmol/l, rise in serum creatinine >30% or fall in creatinine clearance >25%. BVAS: Birmingham vasculitis activity score (v.3); ENT: ear, nose and throat; GPA: granulomatosis with polyangiitis; GFR: glomerular filtration rate; HPF: high-power field; ILD: interstitial lung diseases; MPA: microscopic polyangiitis; SHAP: Shapley additive explanations; XGBoost: extreme gradient boosting

Ensemble clustering identified four distinct clinical phenotypes, designated Clusters A–D (Fig. 2A and B). These clusters were defined by patterns of organ involvement visualized in chord diagrams integrating BVAS organ systems and ILD (Fig. 2C). Cluster A (renal-dominant without ILD) was characterized by renal involvement in all cases and complete absence of ILD. Cluster B (renal-dominant with ILD) comprised patients with concurrent renal involvement and ILD in all cases. Cluster C (systemic multi-organ) showed the broad organ involvement, with frequent general and nervous-system manifestations. Cluster D (ENT-dominant) was defined by predominant ENT involvement and relatively low frequencies of ILD or renal involvement. Clinical profiles of these clusters are summarized in Supplementary Table S4.

Feature-importance analysis using XGBoost with SHAP confirmed these patterns (Supplementary Fig. S1). The top 20 SHAP-ranked features were used to construct the CART model. ILD and renal involvement ranked among the most important features (Fig. 2D), whereas, ‘massive hemoptysis/alveolar hemorrhage’ was infrequent (7.1%) and not included in the top 20 (Supplementary Table S4).

### Cluster reproduction by CART

**Figure 3:**
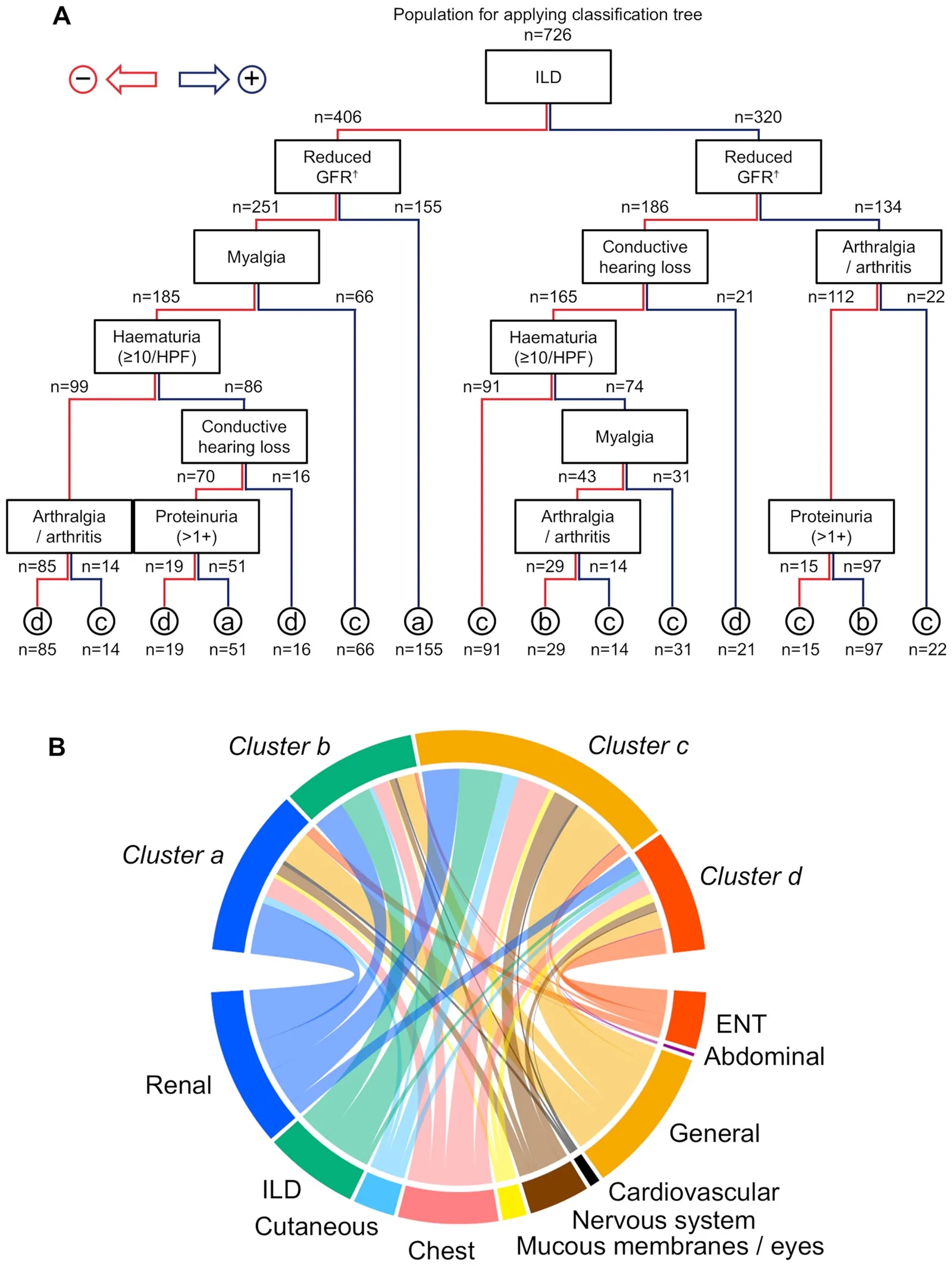
Cluster replication by classification tree. (**A**) Classification tree was constructed using a CART model to replicate ensemble-derived clusters in AAV. The observed number of individuals allocated to each class is shown in each column. (**B**) Chord diagram illustrating the associations of the CART-derived clusters with BVAS organ systems and ILD. Band widths represent the cumulative number of organ involvements for each patient. ☨defined as serum creatinine ≥125 μmol/l, rise in serum creatinine >30% or fall in creatinine clearance >25%. AAV: ANCA-associated vasculitis; CART: classification and regression tree; ENT: ear, nose and throat; GFR: glomerular filtration rate; HPF: high-power field; ILD: interstitial lung diseases

CART model trained on the derivation cohort replicated the four ensemble-derived clusters (Fig. 3), yielding an ARI of 0.72, indicating good concordance.

Application of the CART model separately to the derivation, internal validation and external validation cohorts reproduced clusters exhibiting organ involvement patterns similar to those observed in ensemble clustering (Supplementary Fig. S2).

### Clinical characteristics of the CART-derived clusters

Clinical profiles of the CART-derived clusters, combined the derivation and internal validation cohorts (*n* = 726), are summarized in Table 1 and Supplementary Table S5. When applied to the combined derivation and internal validation cohorts, the CART model reproduced clusters a, b, c and d (Fig. 3).

**Table 1:** Patient characteristics across the CART-derived clusters.

	Total	Clusters			
		Cluster a	Cluster b	Cluster c	Cluster d
	(*n* = 726)	(*n* = 206)	(*n* = 126)	(*n* = 253)	(*n* = 141)
Demographic data					
Age, median [IQR], years	75.00 [68.00, 81.00]	75.00 [69.00, 81.00]	76.00 [69.25, 82.00]	76.00 [69.00, 81.00]	73.00 [66.00, 80.00]
Female, *n* (%)	419 (57.7)	114 (55.3)	62 (49.2)	152 (60.1)	91 (64.5)
MPO-ANCA, *n* (%)	641 (88.3)	183 (88.8)	123 (97.6)	232 (91.7)	103 (73.0)
PR3-ANCA, *n* (%)	96 (13.2)	25 (12.1)	10 (7.9)	25 (9.9)	36 (25.5)
GPA, *n* (%)	173 (23.8)	39 (18.9)	6 (4.8)	41 (16.2)	87 (61.7)
MPA, *n* (%)	553 (76.2)	167 (81.1)	120 (95.2)	212 (83.8)	54 (38.3)
smoking, *n* (%)	422 (58.1)	82 (39.8)	68 (54.0)	104 (41.1)	50 (35.5)
eGFR < 30, *n* (%)	194 (26.7)	107 (51.9)	68 (54.0)	18 (7.1)	1 (0.7)
Comorbidities, *n* (%)					
Hypertension	333 (45.9)	109 (52.9)	71 (56.3)	102 (40.3)	51 (36.2)
Diabetes	155 (21.3)	40 (19.4)	33 (26.2)	50 (19.8)	32 (22.7)
CKD	116 (16.0)	43 (20.9)	38 (30.2)	27 (10.7)	8 (5.7)
Lung disease	235 (32.4)	47 (22.8)	63 (50.0)	87 (34.4)	38 (27.0)
Cardiac disease	110 (15.2)	31 (15.0)	22 (17.5)	47 (18.6)	10 (7.1)
CVD	49 (6.7)	14 (6.8)	13 (10.3)	17 (6.7)	5 (3.5)
Cancer	105 (14.5)	38 (18.4)	23 (18.3)	29 (11.5)	15 (10.6)
FFS ≥ 2	203 (28.0)	99 (48.1)	65 (51.6)	27 (10.7)	12 (8.5)
FFS ≤ 1	523 (72.0)	107 (51.9)	61 (48.4)	226 (89.3)	129 (91.5)
BVAS score, median [IQR]					
Total	15.00 [11.00, 20.00]	16.00 [14.00, 21.00]	17.00 [14.00, 20.75]	13.00 [8.00, 19.00]	11.00 [7.00, 16.00]
BVAS organ systems, *n* (%)					
General	500 (68.9)	137 (66.5)	73 (57.9)	221 (87.4)	69 (48.9)
Cutaneous	141 (19.4)	31 (15.0)	17 (13.5)	66 (26.1)	27 (19.1)
Mucous membrane/eyes	82 (11.3)	17 (8.3)	5 (4.0)	24 (9.5)	36 (25.5)
ENT	194 (26.7)	38 (18.4)	12 (9.5)	45 (17.8)	99 (70.2)
Chest	338 (46.6)	79 (38.3)	65 (51.6)	131 (51.8)	63 (44.7)
Massive haemoptysis/alveolar haemorrhage	47 (6.4)	19 (9.2)	10 (7.9)	15 (5.9)	3 (2.1)
Cardiovascular	35 (4.8)	15 (7.3)	7 (5.6)	10 (4.0)	3 (2.1)
Abdominal	11 (1.5)	1 (0.5)	2 (1.6)	4 (1.6)	4 (2.8)
Renal	539 (74.2)	206 (100.0)	126 (100.0)	148 (58.5)	59 (41.8)
Nervous system	205 (28.2)	47 (22.8)	25 (19.8)	97 (38.3)	36 (25.5)
Other organ involvements, *n* (%)					
ILD	320 (44.1)	0 (0.0)	126 (100.0)	173 (68.4)	21 (14.9)
Hypertrophic pachymeningitis	32 (4.4)	4 (1.9)	3 (2.4)	7 (2.8)	18 (12.8)
Mastoiditis	68 (9.4)	13 (6.3)	4 (3.2)	8 (3.2)	43 (30.5)
Nasal chondritis	13 (1.8)	2 (1.0)	0 (0.0)	6 (2.4)	5 (3.5)
Auricular chondritis	4 (0.6)	1 (0.5)	1 (0.8)	0 (0.0)	2 (1.4)
Remission therapy in the first 2 weeks					
CYC, *n* (%)	200 (27.5)	62 (30.1)	43 (34.1)	61 (24.1)	34 (24.1)
RTX, *n* (%)	157 (21.6)	46 (22.3)	28 (22.2)	53 (20.9)	30 (21.3)
Glucocorticoid therapy					
IVMP, *n* (%)	263 (36.2)	106 (51.5)	64 (50.8)	65 (25.7)	28 (19.9)
Initial PSL, median [IQR] mg	40.00 [30.00, 50.00]	40.00 [35.00, 50.00]	45.00 [40.00, 53.75]	40.00 [30.00, 50.00]	40.00 [30.00, 50.00]

Continuous variables were summarized as median (interquartile range), and categorical variables as *n* (%).

BVAS: Birmingham vasculitis activity score (v.3); CKD: chronic kidney disease; CVD: cerebrovascular disease; ENT: ear, nose and throat; eGFR: estimated glomerular filtration rate; FFS: Five-Factor Score (2009); GPA: granulomatosis with polyangiitis; ILD: interstitial lung diseases; IVCY: intravenous CYC; IVMP: intravenous methylprednisolone; MPA: microscopic polyangiitis; PSL: prednisolone; RTX: rituximab.

As shown in Table 1, Cluster a (renal-dominant without ILD; *n* = 206, 28.4%) and Cluster b (renal-dominant with ILD; *n* = 126, 45.7%) both exhibited multisystem disease with predominant renal involvement, a greater proportion of patients with baseline eGFR <30 ml/min/1.73 m^2^, higher BVAS scores, and higher frequencies of FFS ≥2. Cluster b was further characterized by ILD, a higher prevalence of smoking, and comorbidity with CKD and lung disease. Cluster c (systemic multi-organ; *n* = 253, 34.8%) demonstrated broad organ involvement, including general and nervous-system manifestations, but had less-severe organ involvement and preserved renal function. Cluster d (ENT-dominant; *n* = 141, 19.4%) showed an ENT-dominant pattern, and had the highest proportion of PR3-ANCA positivity (25.5%). Clinical profiles of the CART-derived clusters in the external validation cohort (*n* = 134) showed similar trends (Supplementary Table S6).

The choice of induction therapy aligned with disease severity: patients in Cluster a and Cluster b were more likely to receive CYC (intravenous or oral), with approximately half receiving IVMP pulses, while initial prednisolone doses and RTX use were comparable across clusters.

### Survival and relapse across clusters

**Figure 4:**
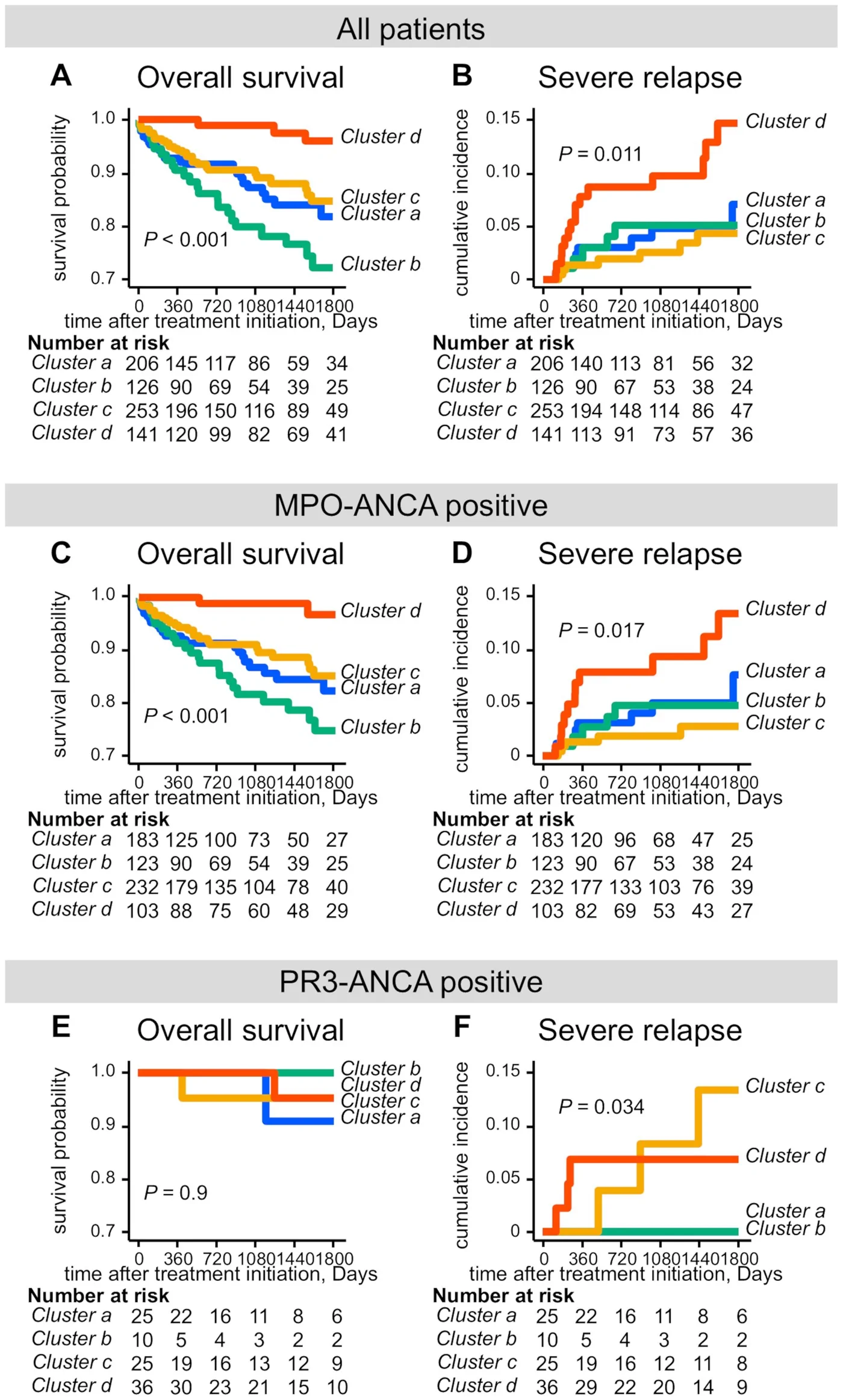
Overall survival and cumulative incidence of relapse according to the clusters. (**A**, **C** and **E**) overall survival; (**B**, **D** and **F**) cumulative incidence of relapse across the CART-derived clusters. (**A** and **B**) all patients; (**C** and **D**) MPO-ANCA-positive patients; (**E** and **F**) PR3-ANCA-positive patients. A log‐rank test was used to compare overall survival. A Gray’s test was performed to compare cumulative incidence of relapse, with death treated as a competing risk. CART: classification and regression tree

Patients from the combined derivation and internal validation cohorts were followed for a median of 2.7 years (IQR, 1.0–4.6). During this period, the cumulative incidence of relapse was 2.8% at 1 year and 4.8% at 5 years, while mortality reached 4.8% at 1 year and 10.5% at 5 years. Overall survival differed among the four clusters (Fig. 4A); patients in Cluster b had the lowest survival, whereas those in Cluster d had the highest. In contrast, the cumulative incidence of relapse was the highest in Cluster d and comparable across Clusters a–c (Fig. 4B).

When stratified by ANCA serotype or disease subtype rather than cluster, overall survival was lower among patients with MPA and those with MPO-ANCA positivity, whereas no clear differences were observed in relapse incidence (Supplementary Fig. S3). Nevertheless, heterogeneity between clusters persisted within the MPO-ANCA-positive subgroup; Cluster b consistently showed the lowest survival, while Cluster d had the highest survival. The cumulative incidence of relapse also remained highest in Cluster d (Fig. 4C and D). In contrast, among PR3-ANCA-positive patients, no clear differences in overall survival were observed across clusters (Fig. 4E), whereas Clusters c and d showed a higher cumulative incidence of relapse (Fig. 4F).

Adjusted survival curves for overall survival and relapse demonstrated favourable survival but a higher incidence of relapse in Cluster d. In contrast, the differences across Clusters a–c were attenuated (Supplementary Figs S4 and S5).

### Treatment responsiveness across clusters

**Figure 5:**
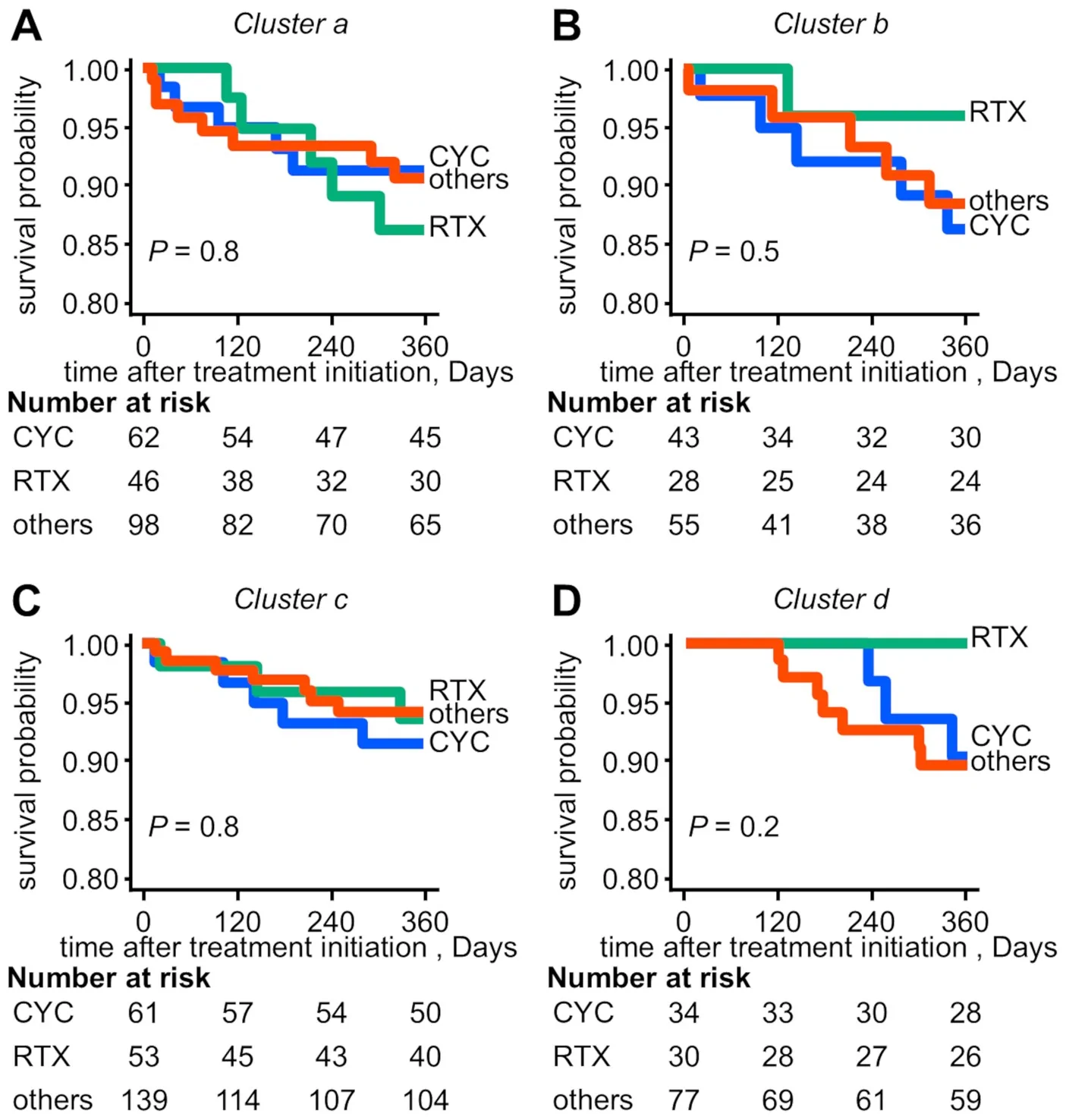
Relapse-free survival according to induction therapy in each cluster. (**A**) Cluster a, (**B**) Cluster b, (**C**) Cluster c and (**D**) Cluster d according to the CART-derived clusters. Each analysis was performed using a log-rank test. Induction therapy was defined as the administration of CYC, RTX or neither within 2 weeks of treatment initiation. CART: classification and regression tree; RTX: rituximab

Patients were stratified by induction therapy initiated within 2 weeks of diagnosis; RTX, CYC (intravenous or oral) or other regimens. Across Cluster a and Cluster c, 1-year relapse-free survival did not differ meaningfully among induction regimens (Fig. 5A and C). Cluster b showed a favourable trend among RTX-treated patients compared with those receiving CYC (Fig. 5B). In Cluster d, patients receiving RTX remained free of death or relapse for 1 year, whereas events occurred in those receiving CYC or other regimens (Fig. 5D). This pattern remained consistent within the MPO-ANCA-positive subgroup (Supplementary Fig. S6).

## Discussion

Using ensemble clustering, we identified four phenotypes with distinct patterns of organ involvement among patients with MPA or GPA: (A) severe renal-dominant disease without ILD; (B) severe renal disease with concurrent ILD; (C) systemic multi-organ disease characterized by broad but generally less-severe organ involvement, including neurologic manifestations and (D) ENT-dominant disease with minimal pulmonary or renal involvement. Using the CART model, these clusters were reproducible in the internal and external validation cohorts, showing good concordance with the ensemble-derived clusters despite differences in cohort characteristics and missing data. Importantly, overall survival and relapse incidence differed between clusters, independent of conventional disease subtype or ANCA serotype. These findings suggest that organ involvement-based phenotyping captures clinically relevant heterogeneity beyond current diagnostic or serologic classifications.

Cluster d (ENT-dominant) exhibited a distinctive pattern of favourable 5-year survival yet the highest incidence of relapse. In this cluster, patients receiving RTX for induction showed potentially superior relapse-free survival compared with those receiving CYC or other regimens. This treatment-response pattern aligned with observations for PR3-ANCA-positive AAV in Western cohorts [6, 7, 21]. Although Cluster d contained a higher proportion of PR3-ANCA-positive patients than the other clusters, the majority was MPO-ANCA-positive. Notably, the trends in survival, relapse and RTX responsiveness remained consistent even when analyses were restricted to MPO-ANCA-positive patients. These findings suggest a subgroup of MPO-ANCA-positive patients exhibiting a PR3-like phenotype, characterized by limited, ENT-dominant organ involvement and susceptibility to relapse despite preserved overall survival.

In contrast, Clusters a–c showed higher mortality but lower relapse rates than Cluster d. Although Cluster a (renal-dominant without ILD) and Cluster b (renal-dominant with ILD) had similarly high BVAS and FFS distributions, Cluster b tended to have poorer survival. The excess mortality in Cluster b may reflect its higher burden of smoking and comorbidities, as the survival difference attenuated after adjustment for these factors. However, smoking and comorbidities may also have contributed to cluster formation. Smoking has been associated with MPO-ANCA-positive AAV, higher disease activity and ILD [22, 23]. Whether genetic susceptibility, sex, lifestyle factors and comorbidities contribute to cluster formation warrants further investigation.

Cluster c (systemic multi-organ) exhibited broad organ involvement, including neurologic manifestations and ILD, a pattern associated with poor outcomes in previous clustering studies [24–26]. Despite less-severe renal disease and lower BVAS/FFS scores than Cluster a, survival and relapse rates were comparable. This may partly reflect the presence of ILD, which is not captured by BVAS/FFS. These findings suggest that BVAS/FFS alone may be insufficient for risk stratification, underscoring the prognostic importance of ILD.

Several previous studies have applied clustering analyses to MPA and GPA, typically identifying 4–7 groups [24–28]. Consistent with these studies, renal involvement was a major contributor to cluster structure in our cohort. In contrast, diffuse alveolar haemorrhage did not define any cluster despite being identified in an earlier cohort [26], likely reflecting its low prevalence (7.1%) [29] in this contemporary cohort. Cluster structures nevertheless varied across studies, likely owing to differences in population background, study era and analytical methodology. More importantly, these findings highlight the considerable clinical heterogeneity of AAV. Although ANCA serotype-based classification is recently considered more predictive of outcomes [6, 7], our findings indicate that organ involvement-based phenotypes provide additional prognostic and predictive information beyond ANCA serotype. Effective patient stratification may therefore require an integrated framework combining both serological and phenotypic dimensions [30].

Unlike previous clustering studies, we treated ILD as a separate variable from other pulmonary manifestations [24, 25]. This distinction is clinically relevant because ILD is highly prevalent in MPO-ANCA-positive MPA, is a major determinant of long-term prognosis and has recently been incorporated into the classification criteria for MPA [5]. Given the predominance of MPO-ANCA positivity and MPA in our cohort, the prominent contribution of ILD to cluster structure is biologically plausible. These findings support the inclusion of ILD in organ involvement-based phenotyping of AAV.

Despite the well-established association between PR3-ANCA positivity and relapse [6, 7], relapse rates did not differ clearly by ANCA serotype in our cohort. This may be attributable to the definition of relapse as major relapse, the limited number of PR3-ANCA-positive patients and more frequent RTX use in PR3-ANCA-positive patients. Together, these factors may have attenuated the expected difference between serotypes.

This study has several limitations. First, the cohort consisted predominantly of Japanese patients, limiting transportability to other populations. Because most patients were MPO-ANCA-positive, the clustering structure may primarily reflect MPO-ANCA-dominant phenotypes, potentially obscuring serotype-specific differences. Second, although clustering variables included extensive organ involvement from the BVAS and the 2022 ACR/EULAR classification criteria, manifestations that were absent or extremely rare could not contribute to cluster formation. Consequently, uncommon but clinically relevant phenotypes may be underrepresented. Third, as this was a retrospective cohort study, treatment strategies were not standardized, although only treatment-naïve patients with newly diagnosed AAV were included. Therefore, the findings from this exploratory analysis should be interpreted cautiously because of potential confounding by indication. Finally, because outcome data were unavailable for the external validation cohort, we could not verify the prognostic heterogeneity observed in the derivation and internal validation cohorts. Future studies using large, multiethnic cohorts with comprehensive follow-up are needed to determine whether these phenotypic clusters and their associations with outcomes are reproducible.

## Conclusion

Through ensemble clustering of organ involvement patterns, we identified four distinct clinical phenotypes among Japanese patients with AAV that differed in prognosis and response to induction therapy. Integrating phenotype-based stratification with ANCA serotype will facilitate individualized risk assessment and therapeutic decision-making. Future integration of cytokine profiling and genetic data may further refine phenotypic classification, improve understanding of disease mechanisms and advance precision medicine in AAV.

## Supplementary Material

keag483_Supplementary_Data
