# Supplementary material for: Clinical phenotypes of ANCA-associated vasculitis using ensemble clustering: prognostic and therapeutic insights from two Japanese cohorts
Source: Rheumatology (Oxford). 2026 Sep 17;65(9):keag483. doi: 10.1093/rheumatology/keag483 (PMC13596050; doi:10.1093/rheumatology/keag483)
Supplement: Supplementary file 1 [file keag483_supplementary_data.docx]

**SUPPLEMENTARY MATERIALS**

Supplementary Figure S1. The top 15 variables contributing to each cluster in the XGBoost model.

Supplementary Figure S2. Chord diagram of the CART-derived clusters with BVAS organ systems and ILD for each cohort.

Supplementary Figure S3. Overall survival and cumulative incidence of relapse according to ANCA serotypes and AAV disease subtypes.

Supplementary Figure S4. Adjusted five-year survival across clusters.

Supplementary Figure S5. Adjusted five-year cumulative incidence of relapse across clusters.

Supplementary Figure S6. Severe relapse-free survival according to induction therapy in MPO-ANCA-positive *Cluster d*.

Supplementary Table S1. Variable preprocessing.

Supplementary Table S2. Characteristics of derivation cohort and internal validation cohorts.

Supplementary Table S3. Characteristics of the external validation cohort.

Supplementary Table S4. Characteristics of the clusters derived from ensemble clustering.

Supplementary Table S5. BVAS scores across the CART-derived clusters.

Supplementary Table S6. Characteristics of external validation cohort clusters patients.

Supplementary Table S7. The list of collaborators of JPVAS.

**Supplementary Figure S1.** The top 15 variables contributing to each cluster in the XGBoost model.

(A) *Cluster A*, (B) *Cluster B*, (C) *Cluster C*, and (D) *Cluster D*.

☨defined as serum creatinine ≥125 μmol/L, rise in serum creatinine >30%, or fall in creatinine clearance >25%.

GFR: glomerular filtration rate; HPF: high-power field; ILD: interstitial lung diseases; XGBoost: extreme gradient boosting

**Supplementary Figure S2.** Chord diagram of the CART-derived clusters with BVAS organ systems and ILD for each cohort.

(A) Derivation cohort, (B) Internal validation cohort, and (C) external validation cohort.

Band widths represent the cumulative number of organ involvements for each patient.

BVAS: Birmingham vasculitis activity score (v.3); CART: classification and regression tree; ILD: interstitial lung diseases.

**Supplementary Figure S3.** Overall survival and cumulative incidence of relapse according to ANCA serotypes and AAV disease subtypes.

(A), (C) Overall survival. (B), (D) Cumulative incidence of severe relapse. (A), (B) Stratified by AAV disease subtypes. (C), (D) Stratified by ANCA serotypes. A log‐rank test was used to compare overall survival. A Gray's test was performed to compare cumulative incidence of severe relapse with death as a competing risk. In this analysis, the 22 patients who were positive for both ANCA types were assigned by the treating physician to either the MPO-ANCA–positive group (n = 19) or the PR3-ANCA–positive group (n = 3).

AAV: ANCA-associated vasculitis; ANCA: antineutrophil cytoplasmic antibody; GPA: granulomatosis with polyangiitis; MPA: microscopic polyangiitis; MPO: myeloperoxidase; PR3: proteinase 3.

**Supplementary Figure S4.** Adjusted five-year survival across clusters.

Five-year survival for *Clusters a–d* adjusted for age, gender, smoking, eGFR < 30, comorbidities (hypertension, diabetes, CKD, lung disease, heart disease, CVD, cancer), disease subtype of AAV, MPO-ANCA, and PR3-ANCA.

AAV: ANCA-associated vasculitis; ANCA: antineutrophil cytoplasmic antibody; CKD: chronic kidney disease; CVD: cerebrovascular disease; eGFR: estimated glomerular filtration rate; MPO: myeloperoxidase; PR3: proteinase 3.

**Supplementary Figure S5.** Adjusted five-year cumulative incidence of relapse across clusters.

Five-year cumulative incidence of relapse with death as a competing risk for *Clusters a–d* adjusted for age, gender, smoking, eGFR < 30, comorbidities (hypertension, diabetes, CKD, lung disease, heart disease, CVD, cancer), disease subtype of AAV, MPO-ANCA, and PR3-ANCA.

AAV: ANCA-associated vasculitis; ANCA: antineutrophil cytoplasmic antibody; CKD: chronic kidney disease; CVD: cerebrovascular disease; eGFR: estimated glomerular filtration rate; MPO: myeloperoxidase; PR3: proteinase 3.

**Supplementary Figure S6.** Severe relapse-free survival according to induction therapy in MPO-ANCA-positive *Cluster d*.

Analysis was performed using a log-rank test. Induction therapy was defined as the administration of CYC, RTX, or neither within two weeks of treatment initiation.

ANCA: antineutrophil cytoplasmic antibody; CYC: cyclophosphamide; MPO: myeloperoxidase; RTX: rituximab.

**Supplementary Table S1.** Variable preprocessing

| BVAS organ systems and　additional variables |  | Variable set 1 |  | Variable set 2 | Variable set 3 |
| --- | --- | --- | --- | --- | --- |
| 1. General | | | | | |
| Myalgia | → | Myalgia | → | Myalgia | General |
| Arthralgia / arthritis | → | Arthralgia / arthritis | → | Arthralgia / arthritis |  |
| Fever ≥38℃ | → | General manifestations | → | General manifestations |  |
| Weight loss ≥2 kg |  |  |  |  |  |
| 2. Cutaneous | | | | | |
| Infarct | → | Ulceration | → | Ulceration | Cutaneous |
| Ulcer |  |  |  |  |  |
| Gangrene |  |  |  |  |  |
| Purpura | → | Purpura | → | Purpura |  |
| Other skin vasculitis | → | Other skin vasculitis | → | Other skin vasculitis |  |
| 3. Mucous membranes /eye | | | | | |
| Mouth ulcers | → | Mouth ulcers | → | Mucous membranes | Mucous membranes /eye |
| Genital ulcers ☨ | → | Genital ulcers ☨ |  |  |  |
| Adnexal inflammation | → | Adnexal inflammation | → | Eyes |  |
| Significant proptosis | → | Significant proptosis |  |  |  |
| Scleritis / Episcleritis | → | Scleritis / Episcleritis |  |  |  |
| Conjunctivitis / Blepharitis / Keratitis | → | Conjunctivitis / Blepharitis / Keratitis |  |  |  |
| Blurred vision | → | Blurred vision |  |  |  |
| Sudden visual loss | → | Sudden visual loss |  |  |  |
| Uveitis | → | Uveitis |  |  |  |
| Retinal changes (vasculitis /thrombosis / exudate /haemorrhage) | → | Retinal changes (vasculitis /thrombosis / exudate /haemorrhage) |  |  |  |
| 4. ENT | | | | | |
| Bloody nasal discharge / crusts /ulcers / granulomata | → | Bloody nasal discharge / crusts /ulcers / granulomata | → | Bloody nasal discharge / crusts /ulcers / granulomata | ENT |
| Paranasal sinus involvement | → | Paranasal sinus involvement | → | Paranasal sinus involvement |  |
| Conductive hearing loss | → | Conductive hearing loss | → | Conductive hearing loss |  |
| Sensorineural hearing loss | → | Sensorineural hearing loss | → | Sensorineural hearing loss |  |
| Subglottic stenosis ☨ | → | Subglottic stenosis ☨ | → | Subglottic stenosis ☨ |  |
| 5. Chest | | | | | |
| Wheeze | → | Wheeze | → | Wheeze | Chest |
| Nodules or cavities | → | Nodules or cavities | → | Nodules or cavities |  |
| Pleural effusion / pleurisy | → | Pleural effusion / pleurisy | → | Pleural effusion / pleurisy |  |
| Infiltrate | → | Infiltrate | → | Infiltrate |  |
| Endobronchial involvement | → | Endobronchial involvement | → | Endobronchial involvement |  |
| Massive haemoptysis / alveolar haemorrhage | → | Massive haemoptysis / alveolar haemorrhage | → | Massive haemoptysis / alveolar haemorrhage |  |
| Respiratory failure | ✕ (nonspecific) | × |  | × |  |
| 6. Cardiovascular | | | | | |
| Loss of pulses ☨ | → | Loss of pulses ☨ | → | Loss of pulses ☨ | Cardiovascular |
| Valvular heart disease | → | Valvular heart disease | → | Cardiovascular |  |
| Pericarditis | → | Pericarditis |  |  |  |
| Ischaemic cardiac pain | → | Ischaemic cardiac pain |  |  |  |
| Cardiomyopathy | → | Cardiomyopathy |  |  |  |
| Congestive cardiac failure | → | Congestive cardiac failure |  |  |  |
| 7. Abdominal | | | | | |
| Peritonitis | → | Peritonitis | → | Abdominal | Abdominal |
| Bloody diarrhoea | → | Bloody diarrhoea |  |  |  |
| Ischaemic abdominal pain | → | Ischaemic abdominal pain |  |  |  |
| 8. Renal | | | | | |
| Hypertension | → | Hypertension | → | Hypertension | Renal |
| Proteinuria >1+ | → | Proteinuria >1+ | → | Abnormal urine test |  |
| Haematuria ≥10 HPF | → | Haematuria ≥10HPFf |  |  |  |
| Serum creatinine 125-249 μmol/L | → | Reduced GFR | → | Reduced GFR |  |
| Serum creatinine 250-499 μmol/L |  |  |  |  |  |
| Serum creatinine ≥500 μmol/L |  |  |  |  |  |
| Rise in serum creatinine >30% or fall in creatinine clearance >25% |  |  |  |  |  |
| 9. Nervous system | | | | | |
| Headache | → | Headache | → | Headache | Nervous system |
| Meningitis | → | Meningitis | → | central nervous system disorder |  |
| Organic confusion | → | Organic confusion |  |  |  |
| Seizures (not hypertensive) | → | Seizures (not hypertensive) |  |  |  |
| Cerebrovascular accident | → | Cerebrovascular accident |  |  |  |
| Cranial nerve palsy | → | Cranial nerve palsy |  |  |  |
| Spinal cord lesion ☨ | → | Spinal cord lesion ☨ |  |  |  |
| Sensory peripheral neuropathy | → | Sensory peripheral neuropathy | → | peripheral neuropathy |  |
| Mononeuritis multiplex | → | Mononeuritis multiplex |  |  |  |
| 10.Others | | | | | |
| Hypertrophic pachymeningitis | → | Hypertrophic pachymeningitis | → | Hypertrophic pachymeningitis | Hypertrophic pachymeningitis |
| ILD | → | ILD | → | ILD | ILD |
| Mastoiditis | → | Mastoiditis | → | Mastoiditis | Mastoiditis |
| Nasal chondritis | → | chondritis | → | chondritis | chondritis |
| Auricular chondritis |  |  |  |  |  |
| 61 variables | → | 53 variables | → | 32 variables | 13 variables |

☨: no cases.

BVAS: Birmingham vasculitis activity score (v.3); ENT: ear, nose, and throat; GFR: glomerular filtration rate; HPF: high-power field; ILD: interstitial lung diseases.

**Supplementary Table S2.** Characteristics of Derivation cohort, Internal validation cohort

|  | Derivation Cohort | Internal validation Cohort | Total |
| --- | --- | --- | --- |
|  | (n = 481) | (n = 245) | (n = 726) |
| Demographic data | | | |
| Age, median [IQR], years | 75.0 [68.0, 81.0] | 75.0 [69.0, 81.0] | 75.0 [68.0, 81.0] |
| Female, n (%) | 268 (55.7) | 151 (61.6) | 419 (57.7) |
| MPO-ANCA, n (%) | 427 (88.8) | 214 (87.3) | 641 (88.3) |
| PR3-ANCA, n (%) | 64 (13.3) | 32 (13.1) | 96 (13.2) |
| ANCA-negative | 7 (1.5) | 4 (1.6) | 11 (1.5) |
| GPA, n (%) | 110 (22.9) | 63 (25.7) | 173 (23.8) |
| MPA, n (%) | 371 (77.1) | 182 (74.3) | 553 (76.2) |
| Time to diagnosis, median [IQR] (days) | 55 [30, 104] | 48 [29, 105] | 53 [30, 104] |
| smoking, n (%) | 264 (54.9) | 158 (64.5) | 422 (58.1) |
| eGFR<30, n (%) | 133 (27.7) | 61 (24.9) | 194 (26.7) |
| Comorbidities, n (%) | | | |
| Hypertension | 223 (46.4) | 110 (44.9) | 333 (45.9) |
| Diabetes | 113 (23.5) | 42 (17.1) | 155 (21.3) |
| CKD | 85 (17.7) | 31 (12.7) | 116 (16.0) |
| Lung disease | 168 (34.9) | 67 (27.3) | 235 (32.4) |
| Cardiac disease | 81 (16.8) | 29 (11.8) | 110 (15.2) |
| CVD | 36 (7.5) | 13 (5.3) | 49 (6.7) |
| Cancer | 69 (14.3) | 36 (14.7) | 105 (14.5) |
| FFS, n (%) | | | |
| FFS >= 2 | 142 (29.5) | 61 (24.9) | 203 (28.0) |
| FFS =< 1 | 339 (70.5) | 184 (75.1) | 523 (72.0) |
| BVAS score, median [IQR] | | | |
| Total | 15.0 [11.0, 20.0] | 14.00 [9.0, 19.0] | 15.0 [11.0, 20.0] |
| General | 2.0 [0.0, 3.0] | 2.0 [0.0, 3.0] | 2.0 [0.0, 3.0] |
| Cutaneous | 0.0 [0.0, 0.0] | 0.00 [0.0, 0.0] | 0.0 [0.0, 0.0] |
| Mucous membrane / eyes | 0.0 [0.0, 0.0] | 0.00 [0.0, 0.0] | 0.0 [0.0, 0.0] |
| ENT | 0.0 [0.0, 2.0] | 0.0 [0.0, 2.0] | 0.0 [0.0, 2.0] |
| Chest | 0.0 [0.0, 4.0] | 0.0 [0.0, 4.0] | 0.0 [0.0, 4.0] |
| Cardiovascular | 0.0 [0.0, 0.0] | 0.0 [0.0, 0.0] | 0.0 [0.0, 0.0] |
| Abdominal | 0.0 [0.0, 0.0] | 0.0 [0.0, 0.0] | 0.0 [0.0, 0.0] |
| Renal | 10.0 [4.0, 12.0] | 10.0 [0.0, 12.0] | 10.0 [0.0, 12.0] |
| Nervous system | 0.0 [0.0, 1.0] | 0.0 [0.0, 1.0] | 0.0 [0.0, 1.0] |
| BVAS organ systems, n (%) | | | |
| General | 322 (66.9) | 178 (72.7) | 500 (68.9) |
| Cutaneous | 91 (18.9) | 50 (20.4) | 141 (19.4) |
| Mucous membrane / eyes | 54 (11.2) | 28 (11.4) | 82 (11.3) |
| ENT | 124 (25.8) | 70 (28.6) | 194 (26.7) |
| Chest | 231 (48.0) | 107 (43.7) | 338 (46.6) |
| Cardiovascular | 23 (4.8) | 12 (4.9) | 35 (4.8) |
| Abdominal | 9 (1.9) | 2 (0.8) | 11 (1.5) |
| Renal | 369 (76.7) | 170 (69.4) | 539 (74.2) |
| Nervous system | 143 (29.7) | 62 (25.3) | 205 (28.2) |
| Other organ involvements, n (%) | | | |
| ILD | 223 (46.4) | 97 (39.6) | 320 (44.1) |
| Hypertrophic pachymeningitis | 22 (4.6) | 10 (4.1) | 32 (4.4) |
| Mastoiditis | 44 (9.1) | 24 (9.8) | 68 (9.4) |
| Nasal chondritis | 7 (1.5) | 6 (2.4) | 13 (1.8) |
| Auricular chondritis | 3 (0.6) | 1 (0.4) | 4 (0.6) |
| Remission therapy in the first 2 weeks | | | |
| CYC, n (%) | 142 (29.5) | 58 (23.7) | 200 (27.5) |
| RTX, n (%) | 76 (15.8) | 81 (33.1) | 157 (21.6) |
| Glucocorticoid therapy | | | |
| IVMP, n (%) | 180 (37.4) | 83 (33.9) | 263 (36.2) |
| Initial PSL, median [IQR] mg | 40.0 [35.0, 50.0] | 40.0 [30.0, 50.0] | 40.0 [30.0, 50.0] |

ANCA: antineutrophil cytoplasmic antibody; BVAS: Birmingham vasculitis activity score (v.3); CKD: chronic kidney disease; CVD: cerebrovascular disease; CYC: cyclophosphamide; ENT: ear, nose, and throat; eGFR: estimated glomerular filtration rate; FFS: five factor score (2009); GPA: granulomatosis with polyangiitis; ILD: interstitial lung diseases; IQR: interquartile range; IVMP: intravenous methylprednisolone; MPA: microscopic polyangiitis; MPO: myeloperoxidase; PSL: prednisolone; PR3: proteinase 3; RTX: rituximab.

**Supplementary Table S3.** Characteristics of the external validation cohort

|  | External validation Cohort |  |
| --- | --- | --- |
|  | (n = 134) | |
| Demographic data |  | |
| Age, median [IQR], years | 75.00 [70.00, 80.00] | |
| Female, n (%) | 90 (67.1) | |
| MPO-ANCA, n (%) | 121 (90.3) | |
| PR3-ANCA, n (%) | 20 (13.4) | |
| ANCA-negative, n (%) | 3 (2.2) | |
| GPA, n (%) | 36 (26.9) | |
| MPA, n (%) | 98 (73.1) | |
| smoking , n (%) | 42 (31.3) | |
| eGFR<30, n (%) | 24 (17.9) | |
| Comorbidities, n (%) |  | |
| Hypertension | 43 (32.1) | |
| Diabetes | 16 (11.9) | |
| CKD | 3 (2.2) | |
| Lung disease | 32 (32.9) | |
| Cardiac disease | 9 (6.7) | |
| CVD | 3 (2.2) | |
| Cancer | 17 (12.9) | |
| FFS, n (%) |  | |
| FFS >= 2 | 37 (27.6) | |
| FFS =< 1 | 97 (72.4) | |
| BVAS score, median [IQR] |  | |
| Total | 17.00 [12.00, 22.00] | |
| General | 2.00 [1.00, 3.00] | |
| Cutaneous | 0.00 [0.00, 0.00] | |
| Mucous membrane / eyes | 0.00 [0.00, 0.00] | |
| ENT | 0.00 [0.00, 2.00] | |
| Chest | 0.00 [0.00, 4.00] | |
| Cardiovascular | 0.00 [0.00, 0.00] | |
| Abdominal | 0.00 [0.00, 0.00] | |
| Renal | 10.00 [6.00, 12.00] | |
| Nervous system | 0.00 [0.00, 1.00] | |
| BVAS organ systems, n (%) |  | |
| General | 104 (77.6) | |
| Cutaneous | 24 (17.9) | |
| Mucous membrane / eyes | 18 (13.4) | |
| ENT | 49 (36.6) | |
| Chest | 59 (44.0) | |
| Cardiovascular | 12 (9.0) | |
| Abdominal | 5 (3.7) | |
| Renal | 107 (79.9) | |
| Nervous system | 35 (26.1) | |
| Other organ involvements, n (%) | | |
| ILD | 73 (54.5) | |
| Hypertrophic pachymeningitis | 5 (3.7) | |
| Mastoiditis | Not collected | |
| Nasal chondritis | Not collected | |
| Auricular chondritis | Not collected | |

ANCA: antineutrophil cytoplasmic antibody; BVAS: Birmingham vasculitis activity score (v.3); CKD: chronic kidney disease; CVD: cerebrovascular disease; CYC: cyclophosphamide; ENT: ear, nose, and throat; eGFR: estimated glomerular filtration rate; FFS: five factor score (2009); GPA: granulomatosis with polyangiitis; ILD: interstitial lung diseases; IQR: interquartile range; IVMP: intravenous methylprednisolone; MPA: microscopic polyangiitis; MPO: myeloperoxidase; PSL: prednisolone; PR3: proteinase 3; RTX: rituximab.

**Supplementary Table S4.** Characteristics of the clusters derived from ensemble clustering

|  | Clustering | | | | Total | |
| --- | --- | --- | --- | --- | --- | --- |
|  | *Cluster A* | *Cluster B* | *Cluster C* | *Cluster D* |  |  |
|  | (n = 124) | (n = 99) | (n = 160) | (n = 98) | (n = 481) | |
| Demographic data | | | | | |  |
| Age, median [IQR], years | 76.0 [69.0, 81.0] | 76.0 [70.0, 82.0] | 75.0 [68.0, 80.0] | 73.0 [66.0, 79.0] | 75.0 [68.0, 81.0] | |
| Female, n (%) | 68 (54.8) | 48 (48.5) | 93 (58.1) | 59 (60.2) | 268 (55.7) | |
| MPO-ANCA, n (%) | 110 (88.7) | 96 (97.0) | 148 (92.5) | 70 (71.4) | 427 (88.8) | |
| PR3-ANCA, n (%) | 11 (8.9) | 3 (3.0) | 11 (6.9) | 25 (25.5) | 64 (13.3) | |
| GPA, n (%) | 20 (16.1) | 6 (6.1) | 23 (14.4) | 61 (62.2) | 110 (22.9) | |
| MPA, n (%) | 104 (83.9) | 93 (93.9) | 137 (85.6) | 37 (37.8) | 371 (77.1) | |
| smoking, n (%) | 53 (42.8) | 55 (55.6) | 71 (44.3) | 38 (38.8) | 217 (45.1) | |
| eGFR < 30, n (%) | 67 (54.0) | 54 (54.5) | 11 (6.9) | 1 (1.0) | 133 (27.7) | |
| Comorbidities, n (%) | | | | | |  |
| Hypertension | 68 (54.8) | 63 (63.6) | 59 (36.9) | 33 (33.7) | 223 (46.4) | |
| Diabetes | 24 (19.4) | 25 (25.3) | 41 (25.6) | 23 (23.5) | 113 (23.5) | |
| CKD | 29 (23.4) | 29 (29.3) | 19 (11.9) | 8 (8.2) | 85 (17.7) | |
| Lung disease | 32 (25.8) | 51 (51.5) | 59 (36.9) | 26 (26.5) | 168 (34.9) | |
| Cardiac disease | 17 (13.7) | 24 (24.2) | 30 (18.8) | 10 (10.2) | 81 (16.8) | |
| CVD | 6 (4.8) | 11 (11.1) | 15 (9.4) | 4 (4.1) | 36 (7.5) | |
| Cancer | 32 (25.8) | 51 (51.5) | 59 (36.9) | 26 (26.5) | 69 (14.3) | |
| FFS, n (%) | | | | | |  |
| FFS >= 2 | 67 (54.0) | 50 (50.5) | 16 (10.0) | 9 (9.2) | 142 (29.5) | |
| FFS =< 1 | 57 (46.0) | 49 (49.5) | 144 (90.0) | 89 (90.8) | 339 (70.5) | |
| BVAS score, median [IQR] | | | | | |  |
| Total | 16.0 [13.0, 20.0] | 16.0 [14.0, 20.0] | 13.0 [9.0, 20.0] | 12.0 [7.0, 18.0] | 15.0 [11.0, 20.0] | |
| General | 2.0 [0.0, 3.0] | 1.0 [0.0, 2.0] | 3.0 [2.0, 3.0] | 2.0 [0.0, 2.0] | 2.0 [0.0, 3.0] | |
| Cutaneous | 0.0 [0.0, 0.0] | 0.0 [0.0,0.0] | 0.0 [0.0, 2.0] | 0.0 [0.0, 0.0] | 0.0 [0.0, 0.0] | |
| Mucous membrane / eyes | 0.0 [0.0, 0.0] | 0.0 [0.0, 0.0] | 0.0 [0.0, 0.0] | 0.0 [0.0, 2.0] | 0.0 [0.0, 0.0] | |
| ENT | 0.0 [0.0, 0.0] | 0.0 [0.0, 0.0] | 0.0 [0.0, 0.0] | 3.0 [0.0, 6.0] | 0.0 [0.0, 2.0] | |
| Chest | 0.0 [0.0, 4.0] | 3.0 [0.0, 4.0] | 4.0 [0.0, 4.0] | 0.0 [0.0, 4.0] | 0.0 [0.0, 4.0] | |
| Cardiovascular | 0.0 [0.0, 0.0] | 0.0 [0.0, 0.0] | 0.0 [0.0, 0.0] | 0.0 [0.0, 0.0] | 0.0 [0.0, 0.0] | |
| Abdominal | 0.0 [0.0, 0.0] | 0.0 [0.0, 0.0] | 0.0 [0.0, 0.0] | 0.0 [0.0, 0.0] | 0.0 [0.0, 0.0] | |
| Renal | 12.0 [10.0, 12.0] | 12.0 [12.0, 12.0] | 4.00 [0.0, 10.0] | 0.0 [0.0, 6.0] | 10.0 [4.0, 12.0] | |
| Nervous system | 0.0 [0.0, 0.0] | 0.0 [0.0, 0.0] | 0.0 [0.0, 6.0] | 0.0 [0.0, 1.0] | 0.0 [0.0, 1.0] | |
| BVAS organ systems, n (%) | | | | | |  |
| General | 75 (60.5) | 53 (53.5) | 139 (86.9) | 55 (56.1) | 322 (66.9) | |
| Cutaneous | 18 (14.5) | 14 (14.1) | 43 (26.9) | 16 (16.3) | 91 (18.9) | |
| Mucous membrane / eyes | 7 (5.6) | 3 (3.0) | 16 (10.0) | 28 (28.6) | 54 (11.2) | |
| ENT | 13 (10.5) | 9 (9.1) | 31 (19.4) | 71 (72.4) | 124 (25.8) | |
| Chest | 48 (38.7) | 53 (53.5) | 92 (57.5) | 38 (38.8) | 231 (48.0) | |
| Massive haemoptysis /  alveolar haemorrhage | 14 (11.3) | 9 (9.1) | 10 (6.3) | 1 (1.0) | 34 (7.1) | |
| Cardiovascular | 11 (8.9) | 6 (6.1) | 6 (3.8) | 0 (0.0) | 23 (4.8) | |
| Abdominal | 1 (0.8) | 3 (3.0) | 3 (1.9) | 2 (2.0) | 9 (1.9) | |
| Renal | 124 (100.0) | 99 (100.0) | 98 (61.3) | 48 (49.0) | 369 (76.7) | |
| Nervous system | 27 (21.8) | 13 (13.1) | 69 (43.1) | 34 (34.7) | 143 (29.7) | |
| Other organ involvement, n (%) | | | | | |  |
| ILD | 0 (0.0) | 99 (100.0) | 110 (68.8) | 14 (14.3) | 223 (46.4) | |
| Hypertrophic pachymeningitis | 2 (1.6) | 2 (2.0) | 5 (3.1) | 13 (14.3) | 22 (4.6) | |
| Mastoiditis | 2 (1.6) | 3 (3.0) | 3 (1.9) | 36 (36.7) | 44 (9.1) | |
| Nasal chondritis | 1 (0.8) | 0 (0.0) | 2 (1.3) | 4 (4.1) | 7 (1.5) | |
| Auricular chondritis | 0 (0.0) | 1 (1.0) | 0 (0.0) | 2 (2.0) | 3 (0.6) | |
| Remission therapy in the first 2 weeks | | | | | |  |
| CYC, n (%) | 36 (29.0) | 35 (35.3) | 42 (26.2) | 29 (29.6) | 142 (29.5) | |
| RTX, n (%) | 17 (13.7) | 17 (17.2) | 28 (17.5) | 14 (14.3) | 76 (15.8) | |
| Glucocorticoid therapy | | | | | |  |
| IVMP, n (%) | 64 (51.6) | 56 (56.6) | 38 (23.8) | 22 (22.4) | 180 (37.4) | |
| Initial PSL, median [IQR] mg | 40.0 [40.0, 50.0] | 45.0 [40.0, 55.0] | 45.0 [30.0, 50.0] | 40.0 [30.0, 50.0] | 40.0 [35.0, 50.0] | |

ANCA: antineutrophil cytoplasmic antibody; BVAS: Birmingham vasculitis activity score (v.3); CKD: chronic kidney disease; CVD: cerebrovascular disease; CYC: cyclophosphamide; ENT: ear, nose, and throat; eGFR: estimated glomerular filtration rate; FFS: five factor score (2009); GPA: granulomatosis with polyangiitis; ILD: interstitial lung diseases; IQR: interquartile range; IVMP: intravenous methylprednisolone; MPA: microscopic polyangiitis; MPO: myeloperoxidase; PSL: prednisolone; PR3: proteinase 3; RTX: rituximab.

**Supplementary Table S5.** BVAS scores across the CART-derived clusters

|  | Clustering | | | | Total |
| --- | --- | --- | --- | --- | --- |
|  | *Cluster a* | *Cluster b* | *Cluster c* | *Cluster d* |  |
|  | (n = 206) | (n = 126) | (n = 253) | (n = 141) | (n = 726) |
| BVAS score, median [IQR] |  |  |  |  |  |
| Total | 16.0 [14.0, 21.0] | 17.0 [14.0, 21.0] | 13.0 [8.0, 19.0] | 11.0 [7.0, 16.0] | 15.0 [11.0, 20.0] |
| General | 2.0 [0.0, 3.0] | 2.0 [0.0, 2.0] | 3.0 [1.0, 3.0] | 0.0 [0.0, 2.0] | 2.0 [0.0, 3.0] |
| Cutaneous | 0.0 [0.0, 0.0] | 0.0 [0.0, 0.0] | 0.0 [0.0, 2.0] | 0.0 [0.0, 0.0] | 0.0 [0.0, 0.0] |
| Mucous membrane / eyes | 0.0 [0.0, 0.0] | 0.0 [0.0, 0.0] | 0.0 [0.0, 0.0] | 0.0 [0.0, 1.0] | 0.0 [0.0, 0.0] |
| ENT | 0.0 [0.0, 0.0] | 0.0 [0.0, 0.0] | 0.0 [0.0, 0.0] | 3.0 [0.0, 6.0] | 0.0 [0.0, 2.0] |
| Chest | 0.0 [0.0, 4.0] | 3.0 [0.0, 4.0] | 3.0 [0.0, 4.0] | 0.0 [0.0, 4.0] | 0.0 [0.0, 4.0] |
| Cardiovascular | 0.0 [0.0, 0.0] | 0.0 [0.0, 0.0] | 0.0 [0.0, 0.0] | 0.0 [0.0, 0.0] | 0.0 [0.0, 0.0] |
| Abdominal | 0.0 [0.0, 0.0] | 0.0 [0.0, 0.0] | 0.0 [0.0, 0.0] | 0.0 [0.0, 0.0] | 0.0 [0.0, 0.0] |
| Renal | 12.0 [10.0, 12.0] | 12.0 [12.0, 12.0] | 4.0 [0.0, 10.0] | 0.0 [0.0, 6.0] | 10.0 [0.0, 12.0] |
| Nervous system | 0.0 [0.0, 0.0] | 0.0 [0.0, 0.0] | 0.0 [0.0, 6.0] | 0.0 [0.0, 1.0] | 0.0 [0.0, 1.0] |

BVAS: Birmingham vasculitis activity score (v.3); CART: classification and regression tree; ENT: ear, nose, and throat; IQR: interquartile range.

**Supplementary Table S6.** Characteristics of external validation cohort clusters patients

|  | Clustering | | | | Total |
| --- | --- | --- | --- | --- | --- |
|  | *Cluster a* | *Cluster b* | *Cluster c* | *Cluster d* |  |
|  | (n = 35) | (n = 29) | (n = 56) | (n = 14) | (n = 134) |
| Demographic data | | | | | |
| Age, median [IQR], years | 77.0 [67.0, 79.0] | 73.0 [70.0, 79.0] | 76.0 [68.0, 84.0] | 74.0 [71.0, 76.0] | 75.0 [70.0, 80.0] |
| Female, n (%) | 24 (68.6) | 14 (48.3) | 43 (76.8) | 9 (64.3) | 90 (67.1) |
| MPO-ANCA, n (%) | 31 (88.6) | 29 (100.0) | 51 (91.1) | 10 (71.4) | 121 (90.3) |
| PR3-ANCA, n (%) | 3 (8.6) | 3 (10.3) | 9 (16.1) | 5 (35.7) | 18 (3.7) |
| GPA, n (%) | 10 (28.6) | 1 (3.4) | 18 (32.1) | 7 (50.0) | 36 (26.9) |
| MPA, n (%) | 25 (71.4) | 28 (96.6) | 38 (67.9) | 7 (50.0) | 98 (73.1) |
| smoking, n (%) | 11 (31.4) | 14 (48.3) | 13 (23.2) | 4 (28.6) | 42 (31.3) |
| eGFR < 30, n (%) | 10 (28.6) | 11 (37.9) | 3 (5.4) | 0 (0.0) | 24 (17.9) |
| Comorbidities, n (%) | | | | | |
| Hypertension | 11 (31.4) | 10 (34.5) | 17 (30.4) | 5 (35.7) | 43 (32.1) |
| Diabetes | 4 (11.4) | 8 (27.6) | 3 (5.4) | 1 (7.1) | 16 (11.9) |
| CKD | 2 (5.7) | 1 (3.4) | 0 (0.0) | 0 (0.0) | 3 (2.2) |
| Lung disease | 5 (14.3) | 9 (31.0) | 16 (28.6) | 2 (14.3) | 32 (32.9) |
| Cardiac disease | 1 (2.9) | 4 (13.8) | 3 (5.4) | 1 (7.1) | 9 (6.7) |
| CVD | 1 (2.9) | 0 (0.0) | 2 (3.6) | 0 (0.0) | 3 (2.2) |
| Cancer | 3 (8.6) | 2 (6.9) | 10 (17.9) | 2 (14.3) | 17 (12.9) |
| FFS, n (%) | | | | | |
| FFS >= 2 | 13 (37.1) | 15 (51.7) | 8 (14.3) | 1 (7.1) | 37 (27.6) |
| FFS =< 1 | 22 (62.9) | 14 (48.3) | 48 (85.7) | 13 (92.9) | 97 (72.4) |
| BVAS score, median [IQR] | | | | | |
| Total | 18.0 [14.0, 22.0] | 18.0 [14.0, 23.0] | 14.0 [10.0, 20.0] | 19.0 [13.0, 24.0] | 17.0 [12.0, 22.0] |
| General | 2.0 [0.0, 3.0] | 2.00 [0.0, 3.0] | 3.0 [2.0, 3.0] | 2.0 [0.5, 3.0] | 2.0 [1.0, 3.0] |
| Cutaneous | 0.0 [0.0, 0.0] | 0.0 [0.0, 0.0] | 0.0 [0.0, 2.0] | 0.0 [0.0, 0.0] | 0.0 [0.0, 0.0] |
| Mucous membrane / eyes | 0.0 [0.0, 0.0] | 0.0 [0.0, 0.0] | 0.0 [0.0, 0.0] | 0.0 [0.0, 3.0] | 0.0 [0.0, 0.0] |
| ENT | 0.0 [0.0, 1.0] | 0.0 [0.0, 2.0] | 0.0 [0.0, 2.0] | 6.0 [5.0, 6.0] | 0.0 [0.0, 2.0] |
| Chest | 0.0 [0.0, 4.0] | 0.0 [0.0, 4.0] | 0.0 [0.0, 4.0] | 2.0 [0.0, 4.0] | 0.0 [0.0, 4.0] |
| Cardiovascular | 0.0 [0.0, 0.0] | 0.0 [0.0, 0.0] | 0.0 [0.0, 0.0] | 0.0 [0.0, 0.0] | 0.0 [0.0, 0.0] |
| Abdominal | 0.0 [0.0, 0.0] | 0.0 [0.0, 0.0] | 0.0 [0.0, 0.0] | 0.0 [0.0, 0.0] | 0.0 [0.0, 0.0] |
| Renal | 12.0 [11.0, 12.0] | 12.0 [12.0, 12.0] | 6.0 [0.0, 10.0] | 6.0 [1.0, 9.0] | 10.0 [6.0, 12.0] |
| Nervous system | 0.0 [0.0, 0.0] | 0.0 [0.0, 0.0] | 0.0 [0.0, 1.0] | 0.0 [0.0, 0.0] | 0.0 [0.0, 1.0] |
| BVAS organ systems, n (%) | | | | | |
| General | 24 (68.6) | 18 (62.1) | 52 (92.9) | 10 (71.4) | 104 (77.6) |
| Cutaneous | 1 (2.9) | 3 (10.3) | 17 (30.4) | 3 (21.4) | 24 (17.9) |
| Mucous membrane / eyes | 3 (8.6) | 1 (3.4) | 8 (14.3) | 6 (42.9) | 18 (13.4) |
| ENT | 9 (25.7) | 8 (27.6) | 18 (32.1) | 14 (100.0) | 49 (36.6) |
| Chest | 16 (45.7) | 10 (34.5) | 26 (46.4) | 7 (50.0) | 59 (44.0) |
| Cardiovascular | 5 (14.3) | 3 (10.3) | 2 (3.6) | 2 (14.3) | 12 (9.0) |
| Abdominal | 2 (5.7) | 1 (3.4) | 2 (3.6) | 0 (0.0) | 5 (3.7) |
| Renal | 35 (100.0) | 29 (100.0) | 33 (58.9) | 10 (71.4) | 107 (79.9) |
| Nervous system | 7 (20.0) | 7 (24.1) | 18 (32.1) | 3 (21.4) | 35 (26.1) |
| Other organ involvement, n (%) | | | | | |
| ILD | 0 (0.0) | 29 (100.0) | 39 (69.6) | 5 (35.7) | 73 (54.5) |
| Hypertrophic pachymeningitis | 1 (2.9) | 0 (0.0) | 3 (5.4) | 1 (7.1) | 5 (3.7) |

ANCA: antineutrophil cytoplasmic antibody; BVAS: Birmingham vasculitis activity score (v.3); CKD: chronic kidney disease; CVD: cerebrovascular disease; CYC: cyclophosphamide; ENT: ear, nose, and throat; eGFR: estimated glomerular filtration rate; FFS: five factor score (2009); GPA: granulomatosis with polyangiitis; ILD: interstitial lung diseases; IVCY: Intravenous cyclophosphamide; IVMP: intravenous methylprednisolone; MPA: microscopic polyangiitis; MPO: myeloperoxidase; PSL: prednisolone; PR3: proteinase 3; RTX: rituximab.

Supplementary Table S7. The list of collaborators of JPVAS

| name | institutional affiliation |
| --- | --- |
| Masao Kikuchi | Division of Cardiovascular Medicine and Nephrology, Department of Internal Medicine, Faculty of Medicine, University of Miyazaki. |
| Hiroshi Akazawa | Department of Cardiovascular Medicine, Graduate School of Medicine, The University of Tokyo. |
| Tatsuya Atsumi | Department of Rheumatology, Endocrinology and Nephrology, Faculty of Medicine and Graduate School of Medicine, Hokkaido University. |
| Tomonori Ishii | Clinical Research, Innovation and Education Center, Tohoku University of Medicine. |
| Tamihiro Kawakami | Division of Dermatology, Tohoku Medical and Pharmaceutical University. |
| Hajime Kono | Department of Internal Medicine, Teikyo University School of Medicine. |
| Yoshinori Matsumoto | Department of Nephrology, Rheumatology, Endocrinology and Metabolism, Faculty of Medicine, Dentistry and Pharmaceutical Sciences, Okayama University. |
| Hiroaki Niiro | Department of Medical Education, Faculty of Medical Sciences, Kyushu University. |
| Natsuka Umezawa | Department of Rheumatology, Graduate School of Medical and Dental Science, Institute of Science Tokyo. |
| Kotaro Matsumoto | Division of Rheumatology, Department of Internal Medicine, Keio University School of Medicine. |
| Toshihiro Nanki | Division of Rheumatology, Department of Internal Medicine, Toho University School of Medicine. |
| Ishizaki Jun | Department of Hematology, Clinical Immunology and Infectious Diseases, Ehime University Graduate School of Medicine. |
| Kan Kishibe | Department of Otolaryngology-Head and Neck Surgery, Asahikawa Medical University. |
| Masashi Bando | Division of Pulmonary Medicine, Department of Medicine, Jichi Medical University. |
| Takao Fujii | Department of Rheumatology and Clinical Immunology, Wakayama Medical University. |
| Yasuhiro Maejima | Department of Cardiovascular Medicine, Graduate School of Medical and Dental Sciences, Institute of Science Tokyo. |
| Hajime Yoshifuji | Department of Rheumatology and Clinical Immunology, Graduate School of Medicine, Kyoto University. |
| Akinori Hara | Department of Nephrology and Rheumatology, Kanazawa University. |
| Mamoru Ayusawa | Department of Pediatrics and Child Health, Nihon University School of Medicine. |
| Shuichi Ito | Department of Pediatrics, Graduate School of Medicine, Yokohama City University. |
| Mitsuyo Itabashi | Department of Nephrology and Dialysis, Tokyo Metropolitan Institute for Geriatrics and Gerontology. |
| Shinya Yamamoto | Department of Nephrology, Graduate School of Medicine, Kyoto University. |
| Masao Ogura | Division of Nephrology and Rheumatology, National Center for Child Health and Development. |
| Toshiaki Usui | Department of Nephrology, Faculty of Medicine, University of Tsukuba. |
| Kazunori Karasawa | Department of Nephrology, Tokyo Women's Medical University. |
| Shoichiro Kanda | Department of Pediatrics, Tokyo University. |
| Takashi Kanda | Department of Clinical Neuroscience, Yamaguchi University Graduate School of Medicine. |
| Masanari Kodera | Department of Dermatology, Japan Community Health Care Organization Chukyo Hospital. |
| Miki Suzuki | Department of Neurology, Tokyo Women's Medical University. |
| Masaya Saito | Department of Hematology, Nephrology, and Rheumatology, Akita University Graduate School of Medicine. |
| Kiyoshi Sekiya | Clinical Research Center for Allergy and Rheumatology, NHO Sagamihara National Hospital. |
| Naoko Nakano | Ehime Prefectural Central Hospital. |
| Hiroko Nagafuchi | Department of Rheumatology and Allergology, St. Marianna University School of Medicine. |
| Hiroshi Nakajima | Department of Allergy and Clinical Immunology, Graduate School of Medicine. |
| Motoshi Hattori | Department of Pediatric Nephrology, Tokyo Women's Medical University. |
| Kunihiro Ichinose | Integrated Kidney Research and Advance, Shimane University Faculty of Medicine. |
| Masahiro Yamamura | Okayama Saiseikai General Hospital. |
| Wako Yumura | Division of Nephrology and Endocrinology, Tohoku Medical and Pharmaceutical University Hospita. |
| Naoko Ishiguro | Department of Dermatology, Tokyo Women's Medical University. |
| Maiko Tanaka | Department of Dermatology, Hiroshima Prefectural Hospital. |
| Shigeru Shibata | Division of Nephrology, Department of Internal Medicine, Teikyo University School of Medicine. |
| Ken-Ei Sada | Department of Clinical Epidemiology, Kochi Medical School. |
| Naotake Tsuboi | Department of Nephrology, Fujita Health University School of Medicine. |
| Naomi Iwata | Aichi Children's Health and Medical Center. |
| Susumu Sakamoto | Department of Respiratory Medicine, Toho University Omori Medical Center. |
| Masami Mizuno | Department of Rheumatology and Clinical Immunology, Gifu University Graduate School of Medicine. |
| Daisuke Kobayashi | Division of Clinical Nephrology and Rheumatology, Graduate School of Medical and Dental Sciences, Niigata University. |
| Hiromichi Tamaki | Immuno-Rheumatology Center, St. Luke's International Hospital |
| Norihiro Kokudo | Hepato-Biliary-Pancreatic Surgery Division, Department of Surgery, National Center for Global Health and Medicine. |
| Aya Kawasaki | Molecular and Genetic Epidemiology Laboratory, Faculty of Medicine, University of Tsukuba. |
| Eisuke Inoue | Showa University Research Administration Center, Showa University. |
| Fumihiko Matsuda | Center for Genomic Medicine, Graduate School of Medicine, Kyoto University. |
| Yoshihisa Yamano | Department of Neurology, St. Marianna University School of Medicine. |
| Shinji Kosugi | Clinical Genetics Unit, Kyoto University Hospital. |
| Motomu Hashimoto | Department of Clinical Immunology, Osaka Metropolitan University Graduate School of Medicine. |

JPVAS: Japan Research Committee of the Ministry of Health
